# Does long‐term soil warming affect microbial element limitation? A test by short‐term assays of microbial growth responses to labile C, N and P additions

**DOI:** 10.1111/gcb.16591

**Published:** 2023-01-19

**Authors:** Chupei Shi, Carolina Urbina‐Malo, Ye Tian, Jakob Heinzle, Steve Kwatcho Kengdo, Erich Inselsbacher, Werner Borken, Andreas Schindlbacher, Wolfgang Wanek

**Affiliations:** ^1^ Division of Terrestrial Ecosystem Research, Department of Microbiology and Ecosystem Science Center of Microbiology and Environmental Systems Science, University of Vienna Vienna Austria; ^2^ Department of Ecosystem and Landscape Dynamics Institute for Biodiversity and Ecosystem Dynamics, University of Amsterdam Amsterdam Netherlands; ^3^ Institute of Soil Science, Leibniz Universität Hannover Hannover Germany; ^4^ Doctoral School in Microbiology and Environmental Science University of Vienna Vienna Austria; ^5^ Department of Forest Ecology and Soil, Federal Research and Training Centre for Forests Natural Hazards and Landscape‐BFW Vienna Austria; ^6^ Department of Soil Ecology, Bayreuth Center of Ecology and Environmental Research (BAYCEER) University of Bayreuth Bayreuth Germany; ^7^ Institute of Soil Research University of Natural Resources and Life Sciences Vienna Austria

**Keywords:** carbon, co‐limitation, long‐term soil warming, microbial growth, nitrogen, nutrient limitation, phosphorus, soil microbes

## Abstract

Increasing global temperatures have been reported to accelerate soil carbon (C) cycling, but also to promote nitrogen (N) and phosphorus (P) dynamics in terrestrial ecosystems. However, warming can differentially affect ecosystem C, N and P dynamics, potentially intensifying elemental imbalances between soil resources, plants and soil microorganisms. Here, we investigated the effect of long‐term soil warming on microbial resource limitation, based on measurements of microbial growth (^18^O incorporation into DNA) and respiration after C, N and P amendments. Soil samples were taken from two soil depths (0–10, 10–20 cm) in control and warmed (>14 years warming, +4°C) plots in the Achenkirch soil warming experiment. Soils were amended with combinations of glucose‐C, inorganic/organic N and inorganic/organic P in a full factorial design, followed by incubation at their respective mean field temperatures for 24 h. Soil microbes were generally C‐limited, exhibiting 1.8‐fold to 8.8‐fold increases in microbial growth upon C addition. Warming consistently caused soil microorganisms to shift from being predominately C limited to become C‐P co‐limited. This P limitation possibly was due to increased abiotic P immobilization in warmed soils. Microbes further showed stronger growth stimulation under combined glucose and inorganic nutrient amendments compared to organic nutrient additions. This may be related to a prolonged lag phase in organic N (glucosamine) mineralization and utilization compared to glucose. Soil respiration strongly positively responded to all kinds of glucose‐C amendments, while responses of microbial growth were less pronounced in many of these treatments. This highlights that respiration–though easy and cheap to measure—is not a good substitute of growth when assessing microbial element limitation. Overall, we demonstrate a significant shift in microbial element limitation in warmed soils, from C to C‐P co‐limitation, with strong repercussions on the linkage between soil C, N and P cycles under long‐term warming.

## Graphical abstract

Increasing global temperatures can differentially affect ecosystem carbon, nitrogen and phosphorous cycling, potentially intensifying elemental imbalances between soil resources, plants and soil microorganisms. Our second longest soil warming project worldwide, the Achenkirch soil warming project, observed that warming had altered microbial element limitation, from carbon limitation to carbon–phosphorous co‐limitation. As soil microbes became nutrient limited, they began to use more C for enzyme production to better acquire the limiting nutrient (phosphorous), resulting in a more wasteful way of C utilization, which caused the forest ecosystem to lose more carbon to the atmosphere.

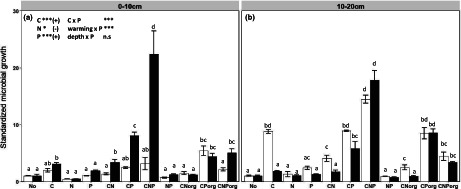

## 1 INTRODUCTION

Temperate forests contain about 14% of the global forest carbon (C) reservoir (Pan et al., 2011) and presently serve as significant atmospheric C sinks. However, despite positive effects on primary production, global warming could upregulate ecosystem respiration to a level where forests may become net sources of CO_2_ (Hadden & Grelle, 2016). Soil microbial communities are among the major players in the terrestrial ecosystem C and nutrient cycles, decomposing soil organic matter (SOM) for their maintenance, growth and enzyme production.

Elevated temperature increases microbial metabolism and enzyme activity (Cookson et al., 2007; Koch et al., 2007), and this has been empirically found to increase SOM decomposition and soil N availability (Bai et al., 2013; Galloway et al., 2004; Hartley et al., 1999; Ma et al., 2011). The improved nutrient availability has been reported to influence the soil C balance via enhanced microbial activity, which can further accelerate soil SOM decomposition and SOM loss (Hartley et al., 2010).

Phosphorus dynamics have only recently received attention in climate change research. In general, temperature can affect soil P availability through its control on soil properties such as pH and SOM content, as well as on plant and microbial activities. For example, warming has been shown to increase available P by accelerating the mineralization of SOM (Liu et al., 2017). However, greater transformation of available P to occluded P was also found under warming (Barrow, 1983; Siebers et al., 2017), which may offset the positive effect of warming on soil available P from accelerated SOM decomposition.

Carbon has been suggested to be the most common limiting element for microbial growth in temperate and boreal forest soils (Alden et al., 2001; Ekblad & Nordgren, 2002; Kamble & Bååth, 2014; Kamble & Bååth, 2016), and soil microbes in a boreal forest were found to be (co)‐limited by N (limited by N or co‐limited by N and other elements) (Kamble & Bååth, 2014). In old tropical soils, microbial (co‐)limitation by P (limitation by P or co‐limitation by P and other elements) is considered as ubiquitous (Camenzind et al., 2018), due to the non‐renewable nature of soil P (Walker & Syers, 1976) and due to the occlusion of soil P through sorption to aluminium and iron oxides in highly weathered environments (Cross & Schlesinger, 1995). Nevertheless, due to human perturbations, atmospheric N emissions are estimated to have increased N deposition significantly compared to preindustrial levels in large areas of Europe (Dentener et al., 2006). This eventually causes forest ecosystems such as at the study site to approach N saturation, as the N inputs (wet and dry N deposition) exceed the outputs (gaseous N emissions; deep percolation) (Herrman et al., 2002). Moreover, decoupling of P cycling from C and N cycling in response to warming has been reported (Geng et al., 2017; Jiao et al., 2016). This could be attributed to the following: (i) The supply of labile C and N is more coupled to each other as labile C and N originate primarily from biological processes, whereas plant‐available P in soil is supplied mainly through abiotic processes, such as mineral weathering and desorption processes (Peter M. Vitousek et al., 2010). (ii) It has long been hypothesized that in contrast to N, organic P mineralization is more decoupled from that of C mineralization (McGill & Cole, 1981). Most organic P compounds in soils are phosphate esters, where P can be cleaved off without necessarily decomposing soil C (Condron et al., 2005). Following the reasoning above, increased SOM decomposition and N mineralization due to warming could make N more available than P, potentially leading to a shift in element(s) that constrain microbial growth.

Microbial element limitation in soils is usually investigated by amending labile substrates to the soil, followed by measuring the response in microbial activity (respiration, enzyme activity, growth) or biomass. Soil enzyme measurements, using enzyme stoichiometry or enzyme vector analysis as proxies of microbial element limitation, reflect the allocation of nutrients by soil microbes toward the production of enzymes mining for elements short in supply. However, soil enzyme stoichiometry has been shown not to reflect microbial element limitation directly (Rosinger et al., 2019). Moreover, respiration is not an ideal proxy to detect limiting elements, because an increase in respiration does not necessarily correspond to increased growth, but merely shows microbial use of C for energy production or overflow respiration (Alden et al., 2001). Growth‐based approaches, therefore, are the gold standard to determine microbial element limitation, by monitoring growth stimulation after adding limiting elements. Radionuclide‐labelled thymidine and leucine incorporation techniques (incorporation of labelled [^3^H] thymidine and [^14^C] leucine into DNA and bacterial protein) have been widely used to estimate microbial growth rates (Bååth, 1998; Demoling et al., 2007; Longnecker et al., 2006). However, some bacteria (e.g. nitrifiers) and most fungi cannot incorporate thymidine (Bloem et al., 2008; Pérez et al., 2010), resulting in an underestimation of microbial growth rates on a community level. For fungi, elemental limitations can, however, be tested independent of bacteria by studying the effect of substrate addition on fungal growth through assessing the ^14^C‐acetate incorporation into ergosterol (Rosinger et al., 2019). In this study, we applied a recently developed approach based on ^18^O incorporation from soil water into genomic microbial DNA (Spohn et al., 2016) to detect limiting elements for microbial growth. Due to the fact that genomic DNA is only synthesized when microbial cells are dividing, microbial growth rates can be calculated based on the incorporation of ^18^O from soil water into DNA (Blazewicz & Schwartz, 2011; Schwartz, 2007; Zheng et al., 2019).

In the present study, we investigated how long‐term soil warming (> 14 years, + 4°C) affected C, N and P limitations of microbial growth and respiration at two soil depths (0–10 cm; 10–20 cm) in a temperate calcareous forest, Achenkirch, Tyrol, Austria. Previous studies found that microbial decomposers in forest soils, even at high soil C:N ratios, are predominantly C limited (Demoling et al., 2007; Kamble et al., 2013; Kamble & Bååth, 2016). Soil warming may further increase microbial C limitation due to the long‐term draw‐down of labile soil C sources. We, therefore, hypothesized that C availability primarily limited microbial growth in all soils and that the warmed soil will become more strongly C‐limited. As the subsoil at the site harbours less labile C reserves (Schindlbacher et al., 2012), we hypothesized a more severe C‐limitation of the subsoil microbial community. Moreover, we investigated whether nutrient quality (addition of organic vs. inorganic N and P) affected the growth response, hypothesizing that organic N and P are preferred as they provide N and P alongside with a source of C and energy, thereby more strongly promoting microbial growth.

## 2 MATERIALS AND METHODS

### 2.1 Site characteristics and soil sampling

In August 2019, soils were sampled in the northern limestone Alps at 910 m a.s.l. in a temperate forest, Achenkirch, Austria (47°34′ 50′′ N; 11°38′ 21′′ E). The dominant tree species was Norway spruce (*Picea abies* (L.) H.Karst.), interspersed by European beech (*Fagus sylvatica* L.) and fir (*Abies alba* Mill.). The soils represent a mosaic of shallow Chromic Cambisols and Rendzic Leptosols, and are characterized by high carbonate contents and near neutral pH. Depth of A‐horizons ranged from 10 to 40 cm, followed by A/C and C horizons of parent dolomite gravel. Fine root biomass was highest in the A‐horizons and decreased with soil depth (Kwatcho Kengdo et al., 2022). Local mean annual air temperature and precipitation were 7°C and 1493 mm (1988–2017) respectively (Achenkirch village, data from ZAMG). The soil warming experiment was set up in 2004 (Schindlbacher et al., 2009). In 2005, soil warming started with three pairs of warmed and adjacent control plots (2 × 2 m each). In 2008, another three paired warmed and control plots were established (Schindlbacher et al., 2009). Soils were warmed (+ 4°C) throughout the snow‐free season (from April/May to December) by inserting resistant heating cables or dummy cables in 3 cm deep slots with a spacing of 7–8 cm in‐between the cable lines.

For the current experiment, we sampled seven to eight soil cores using a soil corer (diameter 2.5 cm, Eijkelkamp, the Netherlands) from each of the six warmed and control plots (*n* = 6) at 0–10 and 10–20 cm soil depth (hereafter topsoil and subsoil). The seven to eight cores of each plot were subsequently pooled and mixed for each sampling depth. From the mixed soil pools of two soil depths and field temperatures, quadruplicate soil samples were taken for the substrate amendment experiment.

### 2.2 Soil preparation

Soils were sieved (<2 mm) and stones and visible roots were removed. Afterwards, soils were transported to the laboratory at the University of Vienna and stored in polyethylene bags at their corresponding field temperature during field sampling, namely 16 and 20°C, for 1 week.

### 2.3 Initial soil physicochemical analyses

Before starting the substrate addition experiment, the soil water content (SWC) was determined by weighing soil aliquots before and after drying at 105°C for 2 days. Water‐holding capacity (WHC) was determined by saturating aliquots of fresh soil (5 g) in a funnel with an ash‐free filter paper, draining the water‐saturated soils for 2.5 h and dividing the water retained in the soils by soil dry mass. As can be seen in Table 2, there were no significant differences between warmed and control soils in terms of initial WHC.

Soil organic C and total soil N were determined by an elemental analyser (Carlo Erba EA1110, Thermo Fisher, USA) after acid pretreatment (2 M HCl) to remove carbonates. Soil total P (TP) was measured in 0.5 M H_2_SO_4_ extracts of ignited (450°C, 4 h) soils by malachite green measurements of reactive phosphate (Robertson et al., 1999). Soil total inorganic P was measured in unignited soils (rest as above), and soil total organic P was estimated by subtraction. Soil pH was determined in a 1:5 (w:v) suspension of air‐dried soil and ultra‐pure water using an ISEFT pH sensor (Sentron, the Netherlands).

### 2.4 Substrate amendment experiment

Substrates were added to quadruplicate soil samples to determine microbial growth‐limiting element(s) in control and warmed soils at their respective mean field temperatures (16°C and 20°C) for both soil depths (0–10 cm; 10–20 cm). To investigate the ‘in situ’ elemental limitations of microbial activities, the incubation temperatures for control and warmed soils were maintained at their respective mean field temperatures. The discussion section goes into greater detail about the choice of incubation temperature.

Carbon (glucose), inorganic N (NH_4_Cl) and inorganic P (KH_2_PO4) were added in a full factorial design. To test nutrient quality (inorganic/organic) effects on microbial growth, glucosamine (contains C and N), glucose‐6‐phosphate (contains C and P) and glucosamine‐6‐phosphate (contains C, N and P) were added to separate soil aliquots. The amount of each substrate added is illustrated in Table 1. To ensure that microbial communities receive comparable amounts of substrates in different soils, the amounts of C added equalled the amount of microbial biomass C in each treatment and at each soil depth. A 100% microbial biomass C was added as a labile C source, as in studies of priming effects (PE, i.e., the mining of soil organic C after activation of the microbial community by labile C addition), where PEs approached zero at this C rate, but increased exponentially above it (Blagodatskaya & Kuzyakov, 2008). When studying microbial C limitation, it is of utmost importance to alleviate microbial C limitation while not triggering positive or negative PEs. The amendments of N and P were set to a specific ratio in relation to the C amendment, that is, to a C:N:P (molar) ratio of 6:1:1, which was constrained by the 6:1:1 C:N:P stoichiometry of glucosamine‐6‐phosphate, and to avoid stoichiometric effects on top of those exerted by the soils themselves.

**TABLE 1:** Substrates added to soils at two field temperatures (control soils: 16°C, warmed soils: 20°C) from two soil depths (0–10; 10–20 cm)

Substrate addition	Total addition (g g^−1^soil C_mic_)		
	C	N	P
No	0	0	0
Glucose	1	0	0
NH_4_Cl	0	0.19	0
KH_2_PO_4_	0	0	0.43
Glucose + NH_4_Cl	1	0.19	0
Glucose + KH_2_PO_4_	1	0	0.43
Glucose + NH_4_Cl + KH_2_PO_4_	1	0.19	0.43
NH_4_Cl + KH_2_PO_4_	0	0.19	0.43
Glucosamine	1	0.19	0
Glucose‐6‐P	1	0	0.43
Glucosamine‐6‐P	1	0.19	0.43

*Note*: Glucose‐carbon (C), NH_4_Cl‐nitrogen (N) and KH_2_PO_4_‐phosphorous (P) were added in a full factorial design. Organic N and P were added as glucosamine (contains C and N), glucose‐6‐P (contains C and P) and glucosamine‐6‐P (contains C, N and P). No indicates the no addition control.

### 2.5 Microbial growth and microbial respiration measurements

Microbial growth rates were measured based on ^18^O incorporation from soil water into genomic DNA (Zheng et al., 2019). ^18^O incorporation into DNA is linear up to 72 h after the addition of ^18^O water at room temperature (Blazewicz & Schwartz, 2011). At intermediate soil temperatures (10–20°C), prolonged time intervals might cause microbial death and turnover, and thereby re‐utilization of ^18^O‐labelled DNA, which would violate the assumptions of such isotope tracing approaches. We therefore restricted the labelling intervals to 24 h, which provides linear kinetics of ^18^O incorporation into genomic DNA without the risk of ^18^O recycling from microbial turnover. All substrates were dissolved in a mixture of H_2_^18^O (97.0 at%, Campro Scientific, Germany) and Milli‐Q water. The ^18^O enrichment and volume of the added H_2_^18^O targeted an at%^18^O enrichment in final soil water of 20 at%^18^O and a final WHC of 70% respectively. Soil aliquots (0.4 g) were weighed into duplicate 2 ml screw cap vials (one for the measurement of growth and respiration by ^18^O‐water addition, one for natural ^18^O abundance (NA) determination of DNA by addition of Milli‐Q water), and similar aliquots were weighed into two sets of 5 ml Greiner tubes (Greiner bio‐one, Cellstar PP, conical) for quantification of microbial biomass and dissolved nutrients. Afterwards, substrate solutions (or Milli‐Q water) were added to the screw cap vials and the Greiner tubes. Directly after substrate addition, the soil aliquots in the 2 ml screw cap vials were transferred to 50 ml glass serum bottles (Supelco, Sigma‐Aldrich Chemie GmbH, Germany) and sealed with a crimp cap and butyl rubber stoppers (Supelco, Sigma‐Aldrich Chemie GmbH, USA). Thereafter, 5 ml headspace gas was sampled immediately by a syringe and measured for CO_2_ concentration by connecting the syringe to an infrared gas analyser (EGM‐4, PP Systems, Amesbury, MA, USA). To keep the environment in the vials at the same atmospheric pressure, 5 ml of air with known CO_2_ concentration was injected back into all vials. Afterwards, all samples were incubated for 24 h at their respective mean field temperatures. At the end of the incubation period (24 h after substrate addition), 5 ml gas sample was collected and determined for CO_2_ concentration using the same infrared gas analyser. The incubation experiment was then stopped by closing the screw cap vials, shock freezing in liquid nitrogen and storage at −80°C until DNA extraction, DNA quantification and oxygen isotope analysis. Total soil DNA was extracted with a DNA extraction kit (FastDNA™ SPIN Kit for Soil, MP Biomedicals, Germany) according to Spohn et al. (2016). DNA concentrations were quantified by the Picogreen fluorescence assay using a microplate spectrophotometer (Infinite M200, Tecan, Austria). Aliquots of DNA extracts were pipetted into silver capsules and dried at 60°C for 2 days. Subsequently, the ^18^O:^16^O isotope composition of soil DNA was analysed using a thermochemical elemental analyser (TCEA) coupled to an isotope ratio mass spectrometer (Delta V Advantage, Thermo Scientific, USA).

The following calculations were performed according to the detailed description in Zheng et al. (2019). Final solution ^18^O enrichment ($\%{\mathrm{at}}_{\text{label}})\;$was calculated by the equation below, where $\%{\mathrm{at}}_{\text{added}}\;$is the at% of the ^18^O‐enriched water added to the soil and *A* is the volume added, *W* is the SWC and $\%{\mathrm{at}}_{\mathrm{NA}}$ is the ^18^O from the NA samples which are close to 0.2 at%:

$$\%{\mathrm{at}}_{\text{label}}=\frac{\%{\mathrm{at}}_{\text{added}}*A+\%{\mathrm{at}}_{\mathrm{NA}}*W}{W+A}$$

The amount of newly produced DNA within 24 h (${\mathrm{DNA}}_{\text{produced}}$) was obtained from the difference in ^18^O abundance between the labelled and the NA samples and using the factor of 31.21, which represents the proportional mass of O in DNA. $O_{\text{Total}}$ is the total O in the dried DNA extracts and $\%{\mathrm{at}}_{\text{excess}}$ is the difference between the at % ^18^O between NA and labelled samples.

$${\mathrm{DNA}}_{\text{produced}}=O_{\text{Total}}\times \frac{\%{\mathrm{at}}_{\text{excess}}}{100}\times \frac{100}{\%{\mathrm{at}}_{\text{label}}}\times \frac{100}{31.21}$$

Microbial growth rates, as microbial C produced by gram of soil dry matter per hour ($C_{\text{growth}}$), were calculated using a conversion factor (fDNA=CmicDNAmic), which describes the relationship between microbial biomass C and microbial DNA content based on soil dry matter, and the quantity of newly produced DNA (Spohn et al., 2016). DW is the soil sample dry weight and *t* is the exact incubation time.

$$C_{\text{growth}}=\left(\frac{\left(\frac{C_{\mathrm{mic}}}{{\mathrm{DNA}}_{\mathrm{mic}}}\right)\times {\mathrm{DNA}}_{\text{produced}}}{\mathrm{DW}\times t}\right)$$

Microbial C uptake was calculated as the sum of mineralized C ($C_{\text{respiration}}$) and C invested into microbial growth ($C_{\text{growth}}$).

$$C_{\text{uptake}}=C_{\text{respiration}}+C_{\text{growth}}$$

Microbial turnover time was calculated as the ratio of microbial biomass C over microbial growth and is therefore given in hours or days.

$$\text{Turnover time}=\frac{C_{\mathrm{mic}}}{C_{\text{growth}}}$$

Basal respiration rates ($C_{\text{respiration}}$) were calculated considering the difference between the initial and final CO_2_ concentration after the incubation period of 24 h. The C respired ($C_{\text{respiration}}$) was calculated per gram dry weight; for further details, please refer to Zheng et al. (2019).

### 2.6 Soil chemical and microbiological analyses after substrate addition

In parallel to microbial growth and respiration analyses, 24 h after substrate addition, one set of soil aliquots in 5 ml Greiner tubes was extracted with 1 M KCl and the other set with 1 M KCl plus 50 μl chloroform (the soil: solution ratio was 1:12.5 (w:v)), which were subsequently put on a shaker for 60 min at 200 rpm and then centrifuged at 10,000 *g* for 5 min. The supernatants were collected and stored in scintillation vials (Sarstedt, Austria) at −20°C for further soil chemical and microbiological analyses. The extraction with 1 M KCl plus chloroform enables the estimation of microbial biomass, similar to the chloroform fumigation extraction (CFE) procedure but is faster (1 h) than CFE (24–48 h). To remove the chloroform, the second set of soil extracts was freeze‐dried for 3 days and then redissolved in the same volume of high purity water. Dissolved organic C (DOC) and total dissolved nitrogen (TDN) were analysed for both sets of KCl extracts using a TOC/TN analyser (TOC‐VCPH Total organic carbon analyser, Shimadzu, Japan). To calculate microbial biomass C (C_mic_) and microbial biomass N (N_mic_), the concentrations of DOC and TDN in the KCl extract without chloroform were subtracted from the concentrations of the soils extracted with KCl and chloroform, and by applying a conversion factor of 2.22 for the calculation according to (Jenkinson, 2004). Ammonium and nitrate in 1 M KCl extracts were quantified by colorimetric methods (Hood‐Nowotny et al., 2010). Acid persulfate digestion (Robertson et al., 1999) was applied to measure total dissolved P (TDP). Dissolved inorganic P (DIP) was determined by applying the malachite green method in 1 M KCl extracts. Soil dissolved organic P (DOP) was calculated as the difference between total dissolved P (TDP) and DIP. Microbial biomass P ($P_{\mathrm{mic}}$) was calculated as the difference in soil TDP between the two sets of KCl extracts, corrected with a conversion factor of 2.5 (Jenkinson, 2004).

### 2.7 Data analyses

Statistical analyses were performed with the R software, version 3.6.1. Data were log‐transformed if necessary, to fit normal distribution of residuals and homogeneity of variance. Overall, only two outliers were identified and substituted by the average value of the other replicates (outliers were tagged if the value was more than 1.5 (IQR) above the upper quartile or more than 1.5 (IQR) below the lower quartile, IQR—interquartile range). Two‐way ANOVA tests were used for testing the effects of warming and soil depth on basic soil physicochemical and microbiological properties in unamended soils. For the main experiments, we applied multifactorial ANOVAs, which seem hard to read, but they allow to test the effect of each factor alone while controlling for all other factors, that is, they test for each main effect after the other main effect in an iterative way, to allow unbiased analysis of single and interactive effects on a response variable (growth response). Five‐way ANOVAs followed by Tukey‐HSD tests were used to test for effects of element addition (C, N and P), soil warming and soil depth on microbial C processes (Table S12). Additionally, six‐way ANOVAs followed by Tukey‐HSD tests were run to test for nutrient quality effects (effects of organic vs. inorganic nutrient additions), that is, for effects of CN, CP and CNP addition, of organic versus inorganic amendment, and effects of soil depth and soil warming on microbial C processes (Table S13). C, N and P amendments were inserted as individual main factors in this ANOVA as, for example, C limitation per se cannot be derived from a statistical design, where all different element additions (C, CP, NP, CNP, etc.) are treated as one main factor and the multiple range tests being not informative. When considering co‐limitation, it is essential to analyse the main effects and the interactions between C, N and P amendments individually and interactively. This is necessary to identify whether the combined addition would be additive or non‐additive (synergistic). The presence or absence of main effects allows to further decipher whether the co‐limitation is related to *simultaneous co‐limitation* (main effects non‐significant, interactive effect positive at *p* < .05) or *independent co‐limitation* (main effects significant at *p* < .05, interactive effect positive at *p* < .05) (Harpole et al., 2011). Moreover, the inclusion of all these main effects of C, N and P, depth and warming and their interactions was important to see whether warming or soil depth altered element limitations.

## 3 RESULTS

### 3.1 Warming and soil depth effects on soil physicochemical and microbiological parameters

Field soil warming decreased soil water content, soil total P, DIP and $P_{\mathrm{mic}}$ (Table 2, *p*‐values of .002, .030, .002 and <.001 respectively), while it slightly decreased DOP (*p* = .050), but increased microbial biomass C:P ratios (*p* < .001). Of these parameters, only $P_{\mathrm{mic}}$ and microbial biomass C:P ratios showed a significant warming × depth interaction, indicating different effect sizes or directions in topsoils and subsoils, here with negative warming effects on $P_{\mathrm{mic}}$ in topsoils, and increases in microbial biomass C:P in response to warming in the subsoil. All other parameters responded similarly to soil warming at both soil depths (no significant warming × depth interaction).

**TABLE 2:** Mean ± standard deviation of soil basic physiochemical and microbiological properties in warmed (20°C) and control (16°C) soil, at soil depths of 0–10 cm and 10–20 cm

x̄ ± STD	Soil depth: 0–10 cm		Soil depth: 10–20 cm		ANOVA test					
	Control	Heated	Control	Heated	Warming		Depth		Warming × depth	
					*p*	*F*	*p*	*F*	*p*	*F*
Water content (% of fresh soil)	53.0% ± 2.3%	41.2% ± 3.4%	40.1% ± 7.5%	31.4% ± 6.2%	**	14.8	**	18.3	n.s	0.4
Initial WHC (%)	44.1% ± 3.5%	44.2% ± 4.6%	49.7% ± 5.3%	48.2% ± 4.5%	n.s	0.7	*	5.6	n.s	0.2
pH	7.1 ± 0.2	6.8 ± 0.3	7.1 ± 0.2	6.8 ± 0.2	*	10.0	n.s	0.4	n.s	0.0
SOC [%]	11.2 ± 1.4	11.1 ± 2.1	7.7 ± 1.4	5.7 ± 2.2	n.s	2.0	***	36.2	n.s	1.6
DOC [μg C g^−1^ DW]	86.7 ± 20.7	66.7 ± 23.4	45.0 ± 15.2	31.7 ± 13.3	***	23.0	n.s	0.3	*	9.0
TDN [μg N g^−1^ DW]	52.3 ± 8.2	44.8 ± 3.3	24.7 ± 1.2	29.4 ± 1.6	n.s	0.3	***	67.4	*	5.4
TN [g kg^−1^]	0.8 ± 0.2	0.8 ± 0.2	0.5 ± 0.1	0.4 ± 0.2	n.s	1.6	***	23.0	n.s	0.0
TP [μg P g^−1^ DW]	792 ± 197	681 ± 222	622 ± 174	408 ± 101	*	4.9	**	9.1	n.s	0.5
Soil C:N ratio	13.7 ± 1.4	15.2 ± 1.3	15.1 ± 1	13.2 ± 0.4	n.s	0.0	n.s	0.7	***	54.3
Nitrate [μg N g^−1^ DW]	31.2 ± 3	35.9 ± 0.9	24.2 ± 0.7	24 ± 0.9	*	7.5	***	131	*	8.7
Ammonium [μg N g^−1^ DW]	6.3 ± 0.3	4.9 ± 0.2	5.7 ± 1	6.3 ± 0.1	n.s	2.1	n.s	2.0	**	13.1
DON [μg N g^−1^ DW]	7.3 ± 4.1	11.4 ± 8.5	0 ± 1.3	0 ± 1.6	n.s ϯ	1.4	** ϯ	23.7	n.s ϯ	0.0
TIP [μg P g^−1^ DW]	76.5 ± 19.2	57.7 ± 16.4	58.8 ± 11.8	44.8 ± 13.8	*	6.6	*	5.8	n.s	0.1
TOP [μg P g^−1^ DW]	715 ± 184	623 ± 207	564 ± 164	370 ± 101	n.s	4.3	**	8.6	n.s	0.6
TDP [μg P g^−1^ DW]	0.9 ± 0	0.8 ± 0	0.7 ± 0	0.6 ± 0	*	5.7	***	53.7	n.s	1.8
DIP [μg P g^−1^ DW]	0.5 ± 0	0.4 ± 0	0.4 ± 0	0.3 ± 0	**	17.6	***	203	n.s	0.2
DOP [μg P g^−1^ DW]	0.1 ± 0	0.1 ± 0	0.1 ± 0	0.1 ± 0	*	5.0	***	19.9	n.s	3.4
C_mic_ [μg C g^−1^ DW]	1080 ± 92	898 ± 42	409 ± 57	529 ± 88	n.s	0.7	***	204	**	17.2
N_mic_ [μg N g^−1^ DW]	122 ± 11	77.5 ± 18.6	51.8 ± 5.2	47.7 ± 8.6	***	42.5	***	304	***	21.5
P_mic_ [μg P g^−1^ DW]	0.7 ± 0	0.5 ± 0	0.3 ± 0	0.2 ± 0	***	59.1	***	329	***	25.4
Molar C_mic_: N_mic_ ratio	10.8 ± 1	12 ± 1	8.6 ± 0.8	13 ± 1.3	***	28.0	n.s	1.1	**	9.6
Molar C_mic:_ P_mic_ ratio	51.3 ± 2	61.9 ± 9.8	50.5 ± 7.6	83.1 ± 10.2	***	28.6	*	6.4	*	7.4
Molar N_mic:_ P_mic_ ratio	4.8 ± 0.3	5.2 ± 0.6	5.9 ± 0.7	6.4 ± 0.4	n.s	3.2	***	21.0	n.s	0.0

*Note*: Stars indicate the results for two‐way ANOVA analysis of the factors warming and soil depth. Note that except for soil pH, soil water content, soil C:N ratio, SOC and TP, all other data were measured parallel to the substrate addition experiment after adjustment of the soil water content to 70% of their water‐holding capacity. Variance homogeneity applied to all data, n.s indicates a non‐significant result, * corresponds to a *p* < .05, ** to a *p* < .01 and *** to a *p* < .001. ϯ Indicates results for log‐transformed data.

In the no‐addition controls, microbial growth rates were higher in topsoil than in subsoil (*p* < .001), showed no general soil warming response (*p* = .36), but inverse responses to warming with depth (*p* interaction of warming × depth = 0.012). In topsoils, warming decreased microbial growth (from 0.39 to 0.25 μg C g^−1^ soil h^−1^), while in subsoils, microbial growth increased (from 0.08 to 0.14 μg C g^−1^ soil h^−1^) (Table S14).

Microbial respiration rates were higher in topsoils than in subsoils (Table S14, *p* < .001). Microbial respiration showed no significant soil warming response (*p* = .10), but similar responses to warming at both soil depths (warming × depth interaction, *p* = .55). Warming tended to increase respiration rates from 2.3 to 2.4 μg C g^−1^ soil h^−1^ and from 0.9 to 1.1 μg C g^−1^ soil h^−1^, in the topsoils and subsoils respectively (Table S14).

### 3.2 Responses of microbial growth to resource amendments

**FIGURE 1:**
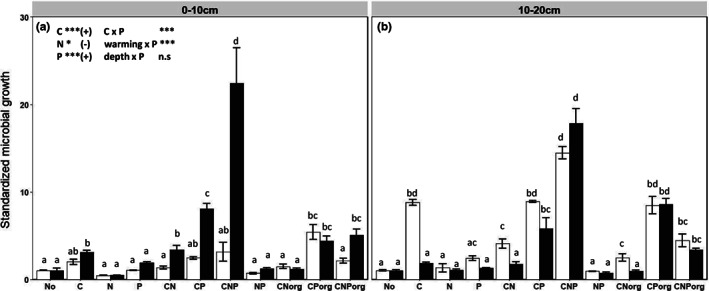
Mean ± standard deviation of standardized soil microbial growth rates 24 h after substrate addition at the respective two field temperatures (control soils: 16°C, white bars; warmed soils: 20°C, black bars) at two soil depths (a: 0–10 cm; b: 10–20 cm). Carbon (C), nitrogen (N) and phosphorus (P) were added in a full factorial design (as C, N, P, CN, CP, NP and CNP). CNorg, CPorg and CNPorg treatments represent the addition of glucosamine, glucose‐6‐P and glucosamine‐6‐P respectively. ‘No’ indicates the no‐addition control. The data were standardized by dividing microbial growth rates of substrate‐amended soils by those of no‐addition controls. Significant main effects from five‐way ANOVA (warming × depth × C × N × P) are given in the graph for *p*‐values <.05 (*), <.01 (**), and <.001 (***). To identify growth‐limiting elements, data for glucose‐C and inorganic nutrient amendments were used for the statistical analyses. Different letters indicated significant differences between growth rates measured in (a) topsoils and (b) subsoils across substrate treatments.

When C, inorganic N or inorganic P was added separately, we found the most pronounced growth stimulation by C amendment. We therefore overall found microbial C limitation as indicated by growth stimulation in C‐amended soils. Growth stimulations did not differ in warmed and control topsoils but decreased with warming in the subsoil (Figure 1, depth × warming interaction, *p* < .001). Control and warmed topsoils showed 2‐ and 3.9‐fold growth stimulation in response to C addition, while in subsoils C addition caused 8.8‐ and 1.8‐fold growth stimulations in control and warmed soils respectively (Figure 1). Nitrogen alone triggered no significant growth response in subsoils, but decreased microbial growth in control and warmed topsoils, though non‐significantly (Figure 1). Phosphorus addition alone tended to stimulate microbial growth in warmed topsoils (*p* < .1), but no P effect was detected in subsoils. These statistical trends were corroborated by a five‐way ANOVAs (warming × depth × C × N × P), showing C to be the primarily limiting element on microbial growth in all soils.

When C and N were added together (CN amendment, significant C × N interaction at *p* < .001, Table S12), microbial growth responses either were not different from C‐only addition (all topsoils and warmed subsoils) or growth decreased (control subsoils, *p* < .05) compared to the C‐only addition. Combined C and P (CP) addition further increased microbial growth significantly in warmed soils (2.6 and 3.2 times greater growth response than in C‐alone additions in topsoil and subsoil respectively, Figure 1), whereas control topsoils were less affected (C × P interaction, *p* < .001; warming × P interaction, *p* = .004). The combined addition of inorganic NP had no significant effect on microbial growth (Figure 1, *p* > .05). Warmed soils showed stronger substrate amendment effects than control soils (*p* < .001), the treatment effects following the order CNP > CP > CN (*p* < .001). These effects were greater in subsoils than in topsoils (*p* < .001). The most pronounced growth stimulation (up to 20‐fold, *p* < .001) was observed following combined C plus N plus P (CNP) addition to warmed soils (Figure 1; C × N × P interaction, *p* < .001).

With regard to the organic versus inorganic nutrient amendments (inorganic/organic here only refers to N and P, as C was always added in organic form as glucose), we found that except for CP_org_ and CP additions, treatments that included inorganic nutrients stimulated microbial growth more strongly than the organic nutrient amendments. This was evident from the significant interaction between type of amendment (CN, CP, CNP) and organic/inorganic origin of the nutrient amendment (*p* < .001). CP_org_ (glucose‐6‐phosphate) and CP (glucose plus inorganic P addition) treatments did not differ significantly in their effect on microbial growth rates (Figure 1). CN_org_ and CNP_org_ addition also triggered growth increases, but to a lower extent compared to the inorganic CN and CNP treatments (Figure 1). The former finding (CP_org_ ~ CP response) was likely caused by the rapid extracellular dephosphorylation of glucose‐6‐P to glucose and free phosphate, causing the CP_org_ treatment therefore closely resembling the CP treatment within short time frames, while such rapid extracellular mineralization of CN_org_ (glucosamine) and CNP_org_ (glucosamine‐6‐phosphate) did not occur. Such differences in extracellular organic N and/or P mineralization were also highlighted in changes in labile N and P fractions (Figures S2 and S3). Most of the glucosamine (CN_org_) added was not mineralized within 24 h, as shown by high DON concentrations and low ammonium and nitrate concentrations (Tables S3 and S4), suggesting that only 21%–32% of glucosamine added was taken up by microbes and mineralized in warmed and control topsoils and subsoils. Notably, glucosamine‐6‐P addition (CNP_org_) stimulated nitrification, as displayed by significant increases in nitrate concentrations compared to no addition controls (Tables S3 and S4; *p* < .001), contributing to higher N mineralization (44%–78%). Comparatively, P mineralization from organic sources was generally greater than that of N. 74%–85% of the added glucose‐6‐P contributed to increased phosphate and P_mic_ concentrations. Glucosamine‐6‐P (CNP_org_) was also rapidly utilized and mineralized, with 66%–88% of added substrate contributing to increased P pools (Tables S5 and S6).

### 3.3 Responses of microbial respiration to resource amendments

**FIGURE 2:**
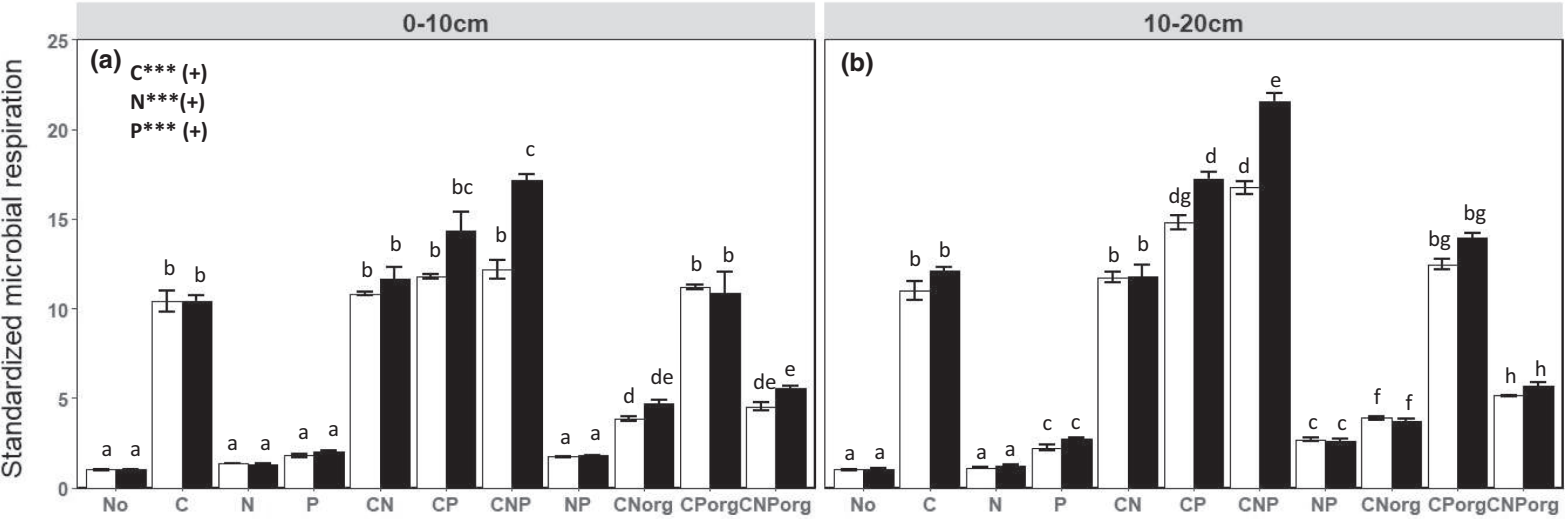
Soil microbial respiration at two field temperatures (control soils: 16°C; heated soils: 20°C) and two soil depths (0–10; 10–20 cm) 24 h after substrate addition. The data were standardized by dividing microbial respiration of amended soils by the value of the no addition control. Significant main effects from five‐way ANOVA (warming × depth × C × N × P) are given in the graph for *p*‐values <.05 (*), <.01 (**) and <.001 (***). Different letters indicated significant differences between respiration rates measured in (a) topsoils and (b) subsoils across substrate treatments.

The responses of soil microbial respiration to substrate additions were similar for warmed and control soils at both soil depths (Figure 2). This was clearly different from the response patterns of microbial growth to substrate amendments, which more strongly differentiated between control and warmed soil microbial communities. The strong positive respiration response upon C addition (*p* < .001) was amplified by CP and CNP amendments (*p* < .001), while N addition alone (*p* > .05) or combined CN addition (*p* < .001) did only weakly stimulate microbial respiration in both treatments (Figure 2). Accordingly, soil microbial respiration was primarily limited by labile C availability and secondarily co‐limited by P. The strong difference in the response to element additions of microbial respiration compared to microbial growth became clearer based on variance partitioning (by calculation of the relative contribution of sums of squares, %SS, of an individual main factor or their interaction to the total sum of squares, from ANOVA analysis). Variance partitioning showed that C amendment explained 28% of the variance in microbial growth, but 87% of that of microbial respiration, indicating very strong differences in the implications of element amendments on growth versus respiratory processes, if used to infer elemental limitations of soil microbial communities. Pearson correlations between the growth and the respiratory responses to substrate additions further highlight the differences in the inference of microbial element limitation based on microbial respiration and growth (*R* = 0.71, *p* < .001, Figure S4). With regard to the inorganic and organic nature of the added substrates, respiration was similarly triggered by CP compared to CP_org_, while CN and CNP treatments had a larger positive effect on respiration compared to CN_org_ and CNP_org_ amendments (Figure 2), similar as became apparent with growth measurements.

### 3.4 Substrate addition effects on labile C, N and P fractions

Soil DOC, which represents one of the most labile soil C pools, increased in all C‐amended soils (Table S7). However, in warmed topsoils, DOC concentrations increased to a lesser extent than in control topsoils. DOC increased the least in the CNP‐treated warmed soils, where microbial C uptake was highest among all amendments (Table S14). Inorganic N and P additions also increased DOC concentrations (Table S7).

P addition alone had no effect on soil nitrate concentrations. However, C addition alone and CP addition decreased soil nitrate concentrations at both soil depths and temperature treatments (Tables S3 and S4).

In both topsoils and subsoils, in all single or combined P treatments, labile P fractions were smaller in warmed soils than in control soils, and more P was routed to the non‐recoverable soil P fraction (Figure S3). Soil warming accelerated abiotic immobilization, which was revealed by higher non‐recoverable P fractions in warmed soils across all treatments (Figure S3).

### 3.5 Substrate addition effects on C_mic_, N_mic_ and P_mic_, and microbial biomass stoichiometry

Substrate addition without C barely affected C_mic_. Based on five‐way ANOVA, we found no N and P effects, but significant positive effects of C (*p* < .001), and negative effects of warming (*p* = .003) and soil depth (*p* < .001) on C_mic_. In more detail, only inorganic CNP additions had a significant positive effect on C_mic_ in warmed topsoils (*p* < .01, Tables S8 and S9; C × N × P interaction, *p* = .003), where C_mic_ increased by a factor of 2.2 compared to no addition controls. Additions of C, N, CN and CP increased C_mic_ in control subsoils, and CN and CNP addition increased C_mic_ in warmed subsoils (Tables S8 and S9). N_mic_ remained stable across all substrate additions and soil depths. The effects of C, N and P additions on P_mic_ were all significant (*p* < .001), and negative warming (*p* = .013) and depth (*p* < .0001) effects were detected. The majority of substrates containing P, either alone or in combination with other elements, increased P_mic_ (with a few exceptions such as CP‐treated topsoils). Substrates containing P decreased C_mic_:P_mic_ and N_mic_:P_mic_ ratios at both temperatures and soil depths (Tables S10 and S11).

## 4 DISCUSSION

### 4.1 Elements limiting microbial growth

Assessments of growth limitations of microbial communities come with several challenges that need to be considered—the choice of incubation temperature, the length of incubation time and the triggering of microbial growth by resource amendments. These (i) differentially affect the underlying processes of microbial growth and (ii) affect the share between the lag phase and the exponential growth phase.

Ad (i): Incubation temperature affects microbial growth rates via the direct control of thermodynamics on molecular interactions in microbial processes (Havird et al., 2020). Many biogeochemical and microbial processes show different temperature sensitivities (Q_10_ values), such as extracellular enzymes, microbial uptake, growth and respiration and diffusion, sorption and desorption processes. Differential temperature sensitivities of microbial and abiotic processes could therefore shift the relative flux partitioning and the constraints on meeting elemental demands by the soil microbial communities and thereby affect elemental limitation of the studied communities when assessed at different temperatures. We therefore here set out to investigate the ‘in situ’ elemental limitations of microbial growth in control and warmed soils, that is, at their in situ temperature, and not to perform a ‘pure’ laboratory assay of microbial processes at the same temperature. The latter, constant temperature, option would provide measures of changes in microbial element limitation unbiased by differences in temperature, which, however, might not be representative for in situ conditions.

Ad (ii): Higher incubation temperatures can reduce the lag phase of microbial growth and proliferation, therefore prolonging the exponential growth phase of microbes. Moreover, when microbes are triggered by labile C addition, the lag phase is reduced at the expense of the prolongation of the exponential growth phase (Nicola & Bååth, 2019; Reischke et al., 2014). Both combined (i.e., warming with C addition) would imply a shorter lag phase of growth in C‐amended warmed soils than in C‐amended control soils, yielding higher estimates of net growth and of growth stimulation by C amendment and implying greater C limitation in warmed soils. First, at the community level, a lag phase does not mean ‘absence of growth’, but only reduced growth through a mix of fewer active and more dormant microbes; this was evident from measurable ^18^O incorporation into microbial DNA in largely inactive microbes under strong drought stress (Canarini et al., 2020). Growth stimulation can therefore be reliably quantified relative to low and basal growth levels. Second, the water‐ (‘No’ control), N‐ and P‐alone additions likely did not change the timing of lag and exponential growth phases in control and warmed soils, as growth was not induced there. Stronger microbial growth only occurred when a pulse of labile C was added, to allow biosynthetic processes and microbial growth. Therefore, the temperature effect on the contribution of exponential growth to the 24‐hour growth period applied only to amendments containing C. In this study, the identification of microbial growth‐limiting elements was achieved by comparing C‐only with C‐plus‐nutrient additions within the same temperature treatment, so that the existence and extent of co‐limitations and changes thereof due to warming and soil depth remain unaffected by different incubation temperatures. Third, our results showed that warmed soils either did not as strongly respond to C addition as control soils (subsoils) or showed similar growth responses to C addition as control soils (topsoils) (Figure 1), instead of showing an expected greater C‐stimulation of growth in warmed soils.

Given the limitations above, the existence of microbial C limitation can be reliably determined at any temperature in comparison to basal growth rates with no amendment, though absolute quantification of the warming effects on the extent of microbial C limitation is not possible. In contrast, given that the labile C amendment determines the timing of lag versus exponential phases, co‐amendment of N and/or P with C is not expected to change this C‐driven timing at one specific temperature, and therefore allow the quantitation of co‐limitations by C and nutrients at any specific temperature.

The significant stimulation of microbial growth (Figure 1) in glucose‐C‐amended treatments compared to the no addition controls reveals that the growth of microbial decomposers at both field temperatures (control and warmed soils) and at both soil depths was primarily limited by a lack of available soil organic C. This confirms the results of previous studies targeting soil microbial element limitation via the assessment of growth stimulation, which showed widespread microbial C limitation (Alden et al., 2001; Demoling et al., 2007; Kamble & Bååth, 2014), though these studies did not assess long‐term soil warming effects. Forests in temperate regions (and in higher latitude ecosystems) are typically thought to be N limited in terms of plant primary production, and soil P availability has been widely viewed to be lower in hot and wet tropical regions than in cold temperate regions, triggering plant P limitation in the tropics (Du et al., 2020; Perakis & Hedin, 2002; Zhang et al., 2005). In contrast to plants, which are well supplied with C by photosynthesis, but usually nutrient (N, P or other) limited, heterotrophic microbes are thought to be primarily limited by C and secondarily by nutrients such as N and P. In addition, first evidence showed that plants and soil microbes do not share the same nutrient (co)limitation (Čapek et al., 2018; Yang et al., 2021). A literature synthesis performed by us (see Data S1) highlights that general trends in P to N (co)limitation of microbial communities in global soils were not apparent with increasing latitude, though the data were scarce, prohibiting statistical data synthesis.

Resource limitation theory has evolved beyond the single nutrient limitation concept (Liebig's Law of the Minimum) to demonstrate widespread co‐limitation by two or more elements (Multiple Limitation hypothesis), either globally (Harpole et al., 2011), or in marine (Fourquez et al., 2020; Mills et al., 2008) and in terrestrial ecosystems (Choi et al., 2022; Ma et al., 2019; Zhang et al., 2015). Harpole et al. (2011) defined strict co‐limitation either as simultaneous or independent co‐limitation. In the case of simultaneous co‐limitation, a positive biomass or growth response only occurs if both resources are added simultaneously, while there is no response in case of single element amendments. In case of independent co‐limitation, positive responses are evident when both nutrients are added individually, but the combined addition commonly produces a super‐additive (synergistic) response. In this study, decadal soil warming had shifted soil microbes from being mainly C limited (control soils) to become strongly CP co‐limited (warmed soils). This becomes clear, as the combined CP amendment stimulated microbial growth by 2.6‐fold to 3.2‐fold beyond that of the C‐only addition. Also, the effect of CP addition on microbial growth was significantly (1.6‐ to 1.9‐fold) stronger than the additive effect of single C and P amendments, and microbial growth showed a positive non‐additive (synergistic) response to combined C and P addition. In warmed topsoils, P addition alone also stimulated microbial growth, while N addition alone had an inhibitory effect on microbial growth. The co‐limitation pattern in warmed soils, therefore, clearly followed independent co‐limitation by C and P sensu Harpole et al. (2011). The shift in microbial growth‐limiting element from C to CP could be due to:

1. Accelerated SOM decomposition in response to warming has preferentially increased the availability of soil N, which has made P relatively less available. McGill and Cole (1981) proposed that N mineralization is more coupled to C mineralization during SOM decomposition. Thus, accelerated microbial processing of SOM, as indicated by the continued ~40% increase of soil CO_2_ efflux to warming (Schindlbacher et al., 2012) at the same site, has led to higher labile N supply (ammonium) in soils (Heinzle et al., 2021) compared to P, leading to elemental imbalances between available soil resources and the soil microbial community.
2. Decadal warming decreased the DOC content, which indirectly lowered soil P availability. A parallel experiment during the same sampling event in this study showed that long‐term warming decreased SOC contents (Tian et al, Nature Communications, in revision), which can affect P supply in two aspects: (i) SOM decomposition releases labile P to the soil solution (ii) and dissolved organic matter releases Pi adsorbed on soil surfaces by competing with them for binding sites (Brucker et al., 2020; Yu et al., 2013). Hence, reduced SOC and DOC contents in warmed soils could indirectly reduce soil P availability through decreased microbially mediated P supply via reduced SOM decomposition and an enhanced Pi sorption capacity of the soil matrix at lower DOC contents.
3. Warming potentially reduced soil P availability through its negative influence on microbial biomass production. It is estimated that P held within soil microorganisms generally accounts for 2%–10% of total soil P (Oberson & Joner, 2005), which represents a significant pool of immobilized P that over longer term through microbial biomass turnover, regulates the P availability in soil solution (Seeling & Zasoski, 1993). Moreover, microbial biomass competes with the soil matrix in immobilizing P, thereby maintaining P in labile forms, which are temporally protected from soil P sorption (Olander & Vitousek, 2004). Hence, reduced C_mic_ due to long‐term soil warming may decrease soil P availability to a certain extent, by shifting soil P cycle controls from microbial toward geochemical P sinks.
4. Warming could have direct negative effects on abiotic Pi supply, by faster transformation of available Pi to occluded P under warming (Barrow, 1983; Siebers et al., 2017). In a parallel experiment at the same site, microbes and plants across all seasons responded to warming by increasing phosphatase production (Tian et al, Nature Communications, in revision). This suggests that soil microbes were investing more energy in organic P acquisition in warmed soils to compensate for reduced abiotic phosphate supply, though microbial P demand in heated soils was not met fully through this supply pathway, as indicated by decreased C_mic_, N_mic_ and P_mic_.

Since soil P availability is less affected by warming‐induced changes in biological processes, but more influenced by abiotic P inputs (Jiao et al., 2016), warming could have negatively affected abiotic P supply pathways. A rise in temperature can directly enhance soil P sorption rates (Barrow, 1983) as well as the migration rate of phosphate into fines pores of hydrous aluminium and iron oxides (Niskanen, 1990), and thereby have dampened abiotic Pi mobilization and reduced P availability in warmed soils. For example, the transformation of secondary P to occluded P is facilitated by warming (Siebers et al., 2017). In our experiment, from the same sampling event as this study, by applying a ^33^P pool dilution method, net abiotic Pi immobilization was accelerated by warming, while inversely microbial P uptake and overall gross Pi mobilization decreased (Tian et al, Nature Communications, in revision). It seems that warming has increased soil Pi sorption (that outcompeted microbial Pi uptake) more than Pi mobilization processes. Moreover, our data show that in all substrate treatments that contained P, the extractable phosphate fraction in warmed soils was smaller than in control soils, and that more P was routed to the non‐recoverable P fraction in warmed soils (Figure S3), while P_mic_ varied little between warmed and control soils or decreased. This provides further evidence that abiotic Pi immobilization through strong sorptive forces was increased by warming, being the main driver causing microbial P co‐limitation.

Experimental soil warming tends to reduce the SWC, as for example, reported in a meta‐analysis of forest soil warming experiments (Xu, Yuan, et al., 2013). However, at this specific site with high annual precipitation, soil rewetting through frequent rainfall events cancelled out such drying effects, and the percentage of WHC during the sampling for this experiment was not different. Drying would decrease substrate diffusion and substrate accessibility in soils (Manzoni & Katul, 2014), as long as drying does not become harmful, and therefore could cause a greater stimulation of growth by substrate addition of starved microbial communities, which may bias this approach toward a greater degree of C limitation or co‐limitation. However, at drier sites or during prolonged dry periods in summer, the drying effect of soil warming may have stronger and negative consequences for soil microbial communities (Schimel, 2018). Dry–rewetting cycles increase substrate availability shortly after rewetting (Borken & Matzner, 2009), likely reducing element limitations in the short term, though under drought growth strongly decreased compared to moist soil conditions (Canarini et al., 2020). In contrast, repeated dry–rewet cycles can cause strong SOC losses, thereby exacerbating microbial C limitations (Malik & Bouskill, 2022). Understanding the effect of soil drying, namely, warming on microbial C limitation is of wider importance, given the increasing incidence of drought periods in a future warmer climate, though it is unlikely that a water deficit will affect the results here in a temperate forest with a mean gravimetric water content of 30%–50% (Davidson et al., 2000).

### 4.2 Microbial use of organic nutrients

Lower stimulation of microbial C uptake and growth in CN_org_‐ and CNP_org_‐treated soils compared to CN and CNP treatments, but equivalent stimulation of C uptake and growth by CP_org_ and CP additions shows a lower degree of microbial utilization of glucosamine and glucosamine‐6‐phosphate than of glucose and glucose‐6‐P. This indicates that organic nutrients (providing organic C and nutrients in one) are not necessarily preferred over inorganic nutrients plus a C source. As shown by Roberts et al. (2007), microbial mineralization of glucose peaked at around 24 h after substrate amendment, regardless of substrate concentration. In contrast, the glucosamine mineralization rate reached its peak around 48 h after substrate addition. Slow mineralization of glucosamine to ammonium and glucose (Figure S2) and/or low expression levels of specific carrier proteins for intact uptake of this organic N form relative to glucose and ammonium transporters likely triggered these lags of glucosamine use. Greater organic N mineralization was found in the glucosamine‐6‐P treatment, probably due to alleviation of P limitation and stimulation of glucosamine‐6‐P mineralization, which has also made more C and P available for microbial uptake and growth. This lends strong support to P mineralization being mainly controlled by microbial P demand (McGill & Cole, 1981), instead of being activated by the microbial need for C in C‐ and P‐limited soils (Heuck et al., 2015; Spohn & Kuzyakov, 2013). The latter hypothesis is also rather unlikely due to the rarity of organic P (CP_org_) compounds relative to C_org_ compounds being devoid of N and P. Based on the global average soil C:N:P (molar 287:17:1, Xu, Thornton, & Post, 2013) and a typical organic C compound with 6 C atoms (and a C:N:P ratio of 6:1:1), a microbe would only find one molecule CP_org_ under 50 C_org_ molecules in SOM to ameliorate microbial C limitation and one molecule CP_org_ under 500–700 C_org_ molecules in leaf litter and roots.

### 4.3 Substrate addition effects on microbial biomass C:N:P stoichiometry

Changes in microbial biomass C:N:P stoichiometry upon element amendments are due to microbial uptake of C, N or P in excess and storage of these elements by microbes. Excess microbial P uptake could explain the increase in P_mic_ with no corresponding increase in C_mic_, excess microbial P being stored in the form of polyphosphates. Since glucose is readily available for microbial uptake, it was most likely used by microbes to build up C storage molecules such as polyhydroxybutyrates or was invested into growth, both resulting in higher C_mic_ in some of the C‐treated soils. Considering changes in microbial community structure, R‐strategists can take up high amounts of P (disproportionally to C and N) to synthesize ribosomal RNA (Elser et al., 2000), and therefore, an activation of R‐strategists could explain higher microbial growth rates in warmed compared to control soils upon addition of P‐containing substrates. This is to some extent supported by our data, showing simultaneous increases of P_mic_ and decreases of C_mic_:P_mic_ and N_mic_:P_mic_ ratios in both control and warmed soils, when P‐containing substrates were added. In contrast, Rhodospirillales, known copiotrophs (R‐strategists), were less abundant in the warmed soils at the study site (Liu et al., 2017), indicating greater dominance of oligotrophs. Since oligotrophic microbial species can still grow when exposed to limited C and nutrient supplies, while copiotrophic organisms are specialized in thriving on high C concentrations under optimal nutrient conditions (Fierer et al., 2007), a taxonomic shift from copiotrophic‐ to oligotrophic‐dominated communities might have occurred under prolonged warming. However, a shift in microbial community structure (copiotrophic to oligotrophic or bacteria to fungal dominance) likely was not the dominant explanatory factor for the observed warming effects. Our results also displayed homeostatic behaviour of soil microbes under nutrient amendments without P, but non‐homeostatic behaviour of soil microbes when receiving P amendments in both control (mainly C‐limited) and warmed soils (CP co‐limited). Considering the prevailing microbial element limitations at the study site, it can therefore be argued that in temperate forest soils, microbes suffering from CP co‐limitation, P addition will stimulate microbial P uptake in excess, which could subsequently increase P_mic_ and decrease microbial biomass C:P and N:P ratios.

## 5 CONCLUSION

Decadal forest soil warming has altered microbial growth‐limiting elements, from C limitation to CP co‐limitation. While N is supplied rapidly enough through SOM decomposition in warmed soils, P supply via weathering and desorption/dissolution pathways did not keep up. In contrast, abiotic sorption of P increased in warmed soils, decreasing soil P availability and triggering microbial CP co‐limitation. P (co‐)limitation likely shifts the resource allocation strategy of soil microbes ever more from high yield to resource acquisition, for example, by increasing phosphatase production and secretion. This has significant implications for ecosystem C storage, since microbes would allocate more C to energy and enzyme production, resulting in a decreased microbial C use efficiency and reduced growth, thus reducing the C storage capacity of forest ecosystem. Moreover, warming induced increases in fine root biomass and production (Kwatcho Kengdo et al., 2022), and therefore, intensified plant‐derived C input into the belowground compartment will likely not result in greater microbial necromass and SOM formation. At the study site, decreased DOC and SOC have already been observed, suggesting ecosystem net C losses regardless of the increased C inputs from plants. Given that tree species across Europe show increasing signs of P limitation during the last decades (Du et al., 2021), warming might even more strongly affect the overall performance and C sequestration potential of temperate forests. The interaction of warming × P limitation has largely remained elusive and therefore strongly reduces our capability to understand the biogeochemical future of forests.

## CONFLICT OF INTEREST

The authors declare no competing interests.

## Supporting information


Data S1.

## Data Availability

The data that support the findings of this study are openly available in DRYAD at https://doi.org/10.5061/dryad.41ns1rnjd
