# Supplementary material for: Does long‐term soil warming affect microbial element limitation? A test by short‐term assays of microbial growth responses to labile C, N and P additions
Source: Glob Chang Biol. 2023 Jan 19;29(8):2188–202. doi: 10.1111/gcb.16591 (PMC10946488; doi:10.1111/gcb.16591)
Supplement: Supplementary file 1 — Data S1. [file GCB-29-2188-s001.docx]

## **Supplement**

**Does long-term soil warming affect microbial element limitation? A test by short-term assays of microbial growth responses to labile C, N and P additions**

Chupei Shi, Carolina Urbina-Malo, Ye Tian, Jakob Heinzle, Steve Kwatcho Kengdo, Erich Inselsbacher, Werner Borken, Andreas Schindlbacher, Wolfgang Wanek

### **Supplementary text**

Measurements of microbial growth responses (stimulation) to factorial C, N and P additions are certainly the most direct method to assess element limitation of soil microbes, as it has been done in experimental manipulations at the whole ecosystem level assessing plant/vegetation growth responses to N, P or other nutrient additions (P. M. Vitousek, 2004). We carefully reviewed and analyzed the literature on growth-based element limitation studies of soil microbes and considered any response greater +25% growth stimulation as indicative of microbial element limitation. This data synthesis (Table S2 included tropical forests and pastures (C. Rosinger, Rousk, & Sanden, 2019) and tropical cropland soils (P. N. Kamble, Gaikwad, Kuchekar, & Bååth, 2014), temperate forests (P. N. Kamble & Bååth, 2014, 2016; P. N. Kamble, Rousk, Frey, & Bååth, 2013) and subalpine grasslands and shrublands (Goransson, Venterink, & Bååth, 2011), and boreal garden, cropland, managed grassland and forest soils (Demoling, Figueroa, & Bååth, 2007). Compiling these data showed the following patterns: Fungal growth was rarely measured (four studies, five soils) and only in one out of the five investigated soils (a Swedish garden soil) fungal growth was stimulated by any C-N-P amendment, and here upon C and C+N addition. The responsiveness of bacterial growth to any of the C-N-P additions was maximal in the tropics (100% of all cases) and decreased towards temperate and boreal ecosystems (84-93%), as indicated by increases in no responses (“NO”) in the Table below (Table S1, Figure S1).

**Table S1:** Number of observations and percentages of bacterial growth stimulation by factorial C, N and P amendments used in this meta-analysis. Soil bacterial growth limitation was assessed by positive responses to the addition of labile C, N or P, and their combinations. Top – absolute numbers, bottom - the percentage of soils showing a positive response are presented, where a positive response was defined as >25% increase in growth relative to controls with no addition of C, N or P. C+ includes any soil responding to C amendment, independent whether other nutrients were provided in combination with C. C+ only represents soils that only responded posititive to C addition alone, but did not show any other response to other element additions. NO depicts the percentage of soils being unresponsive to the amendments.

**Figure S1:** Percentage of soils where bacterial growth responded positively to different nutrient additions.

Across all 66 tested soils bacterial growth only each once showed positive responses to single N or P additions (two boreal agricultural soils, see Table S2), otherwise N frequently negatively affected bacterial growth while P effects were neutral. All other soils showed positive effects of short-term C or C-nutrient additions, indicating that soil bacterial growth was mainly C/energy limited or C-N, C-P or C-N-P co-limited. There were systematic differences in this pattern across different ecosystems and biomes. Single microbial C limitation peaked in boreal soils (36%), was lower for tropical soils (23%) and lowest in temperate soils (4%). Bacterial C-N co-limitation was higher in tropical and temperate systems (62-72%) than in boreal ones (43%), while C-N-P co-limitation peaked in the tropics. C-P co-limitation was greatest in temperate (mostly subalpine) ecosystems (72%). There was evidence that land use (contrast only for boreal ecosystems; forests versus agricultural land which included gardens, managed grasslands and croplands) affected soil bacterial growth limitation. Bacterial growth was more strongly limited by C-only in boreal forest soils (43%) than in boreal agricultural soils (29%). In agricultural soils C-N (64%) and C-N-P co-limitation (21%) was elevated relative to forest soils (21% and 0%, respectively), while in forest soils a higher frequency of C-P co-limitation (21%) was evident compared to agricultural soils. We can also compare tropical agricultural soils (P. N. Kamble et al., 2014; C. Rosinger, Rousk, & Sanden, 2019) with boreal agricultural soils (Demoling, Figueroa, & Bååth, 2007). This analysis showed similar levels of bacterial C limitation (86-100%), C-only limitation (25-29%) and C-N co-limitation (64-67%), but stronger C-N-P co-limitation in tropical agricultural soils. When only considering (semi)natural ecosystems we can compare temperate grasslands, shrublands and forests (Goransson et al., 2011; P. N. Kamble & Bååth, 2014, 2016; P. N. Kamble et al., 2013) with those of boreal forests in Sweden (Demoling et al., 2007). Here C-only limitation of soil bacteria increased from temperate to boreal ecosystems (from 4 to 43%), while bacterial C-N and C-P co-limitation decreased from temperate to boreal woody ecosystems (72 to 21% each). This data synthesis therefore shows an increasing microbial C limitation with latitude and decreasing microbial N (co)limitation with latitude. In N limited hardwood forests in N-America microbes were mainly C limited (Aber et al., 1993) and particularly positively responded to combined C-N addition (indicating C-N co-limitation), whilst showing an increasing growth response to C with increasing N fertilizer rate (P. N. Kamble et al., 2013). This clearly indicates that plants and microbes may be limited by different elements.

**Table S2**: Literature sources for the data synthesis on microbial (bacterial and fungal) growth limitation.

**Supplementary references**

Aber, J. D., Magill, A., Boone, R., Melillo, J. M., Steudler, P., & Bowden, R. (1993). Plant and soil responses to chronic nitrogen additions at the Havard Forest, Massachusetts. *Ecological Applications, 3*(1), 156-166. <http://doi.org/10.2307/1941798>

Demoling, F., Figueroa, D., & Bååth, E. (2007). Comparison of factors limiting bacterial growth in different soils. *Soil Biology and Biochemistry, 39*(10), 2485-2495. http://doi.org/10.1016/j.soilbio.2007.05.002

Goransson, H., Venterink, H. O., & Bååth, E. (2011). Soil bacterial growth and nutrient limitation along a chronosequence from a glacier forefield. *Soil Biology and Biochemistry, 43*(6), 1333-1340. http://doi.org/10.1016/j.soilbio.2011.03.006

Kamble, P. N., & BÅÅTh, E. (2018). Carbon and Nitrogen Amendments Lead to Differential Growth of Bacterial and Fungal Communities in a High-pH Soil. *Pedosphere, 28*(2), 255-260. http://doi.org/10.1016/s1002-0160(18)60014-1

Kamble, P. N., & Bååth, E. (2016). Comparison of fungal and bacterial growth after alleviating induced N-limitation in soil. *Soil Biology and Biochemistry, 103*, 97-105. http://doi.org/10.1016/j.soilbio.2016.08.015

Kamble, P. N., & Bååth, E. (2014). Induced N-limitation of bacterial growth in soil: Effect of carbon loading and N status in soil. *Soil Biology and Biochemistry, 74*, 11-20. http://doi.org/10.1016/j.soilbio.2014.02.015

Kamble, P. N., Gaikwad, V. B., Kuchekar, S. R., & Bååth, E. (2014). Microbial growth, biomass, community structure and nutrient limitation in high pH and salinity soils from Pravaranagar (India). *European Journal of Soil Biology, 65*, 87-95. http://doi.org/10.1016/j.ejsobi.2014.10.005

Kamble, P. N., Rousk, J., Frey, S. D., & Bååth, E. (2013). Bacterial growth and growth-limiting nutrients following chronic nitrogen additions to a hardwood forest soil. *Soil Biology and Biochemistry, 59*, 32-37. http://doi.org/10.1016/j.soilbio.2012.12.017

Rosinger, C., Rousk, J., & Sanden, H. (2019). Can enzymatic stoichiometry be used to determine growth-limiting nutrients for microorganisms? - A critical assessment in two subtropical soils. *Soil Biology and Biochemistry, 128*, 115-126. http://doi.org/10.1016/j.soilbio.2018.10.011

### **Supplementary figures**


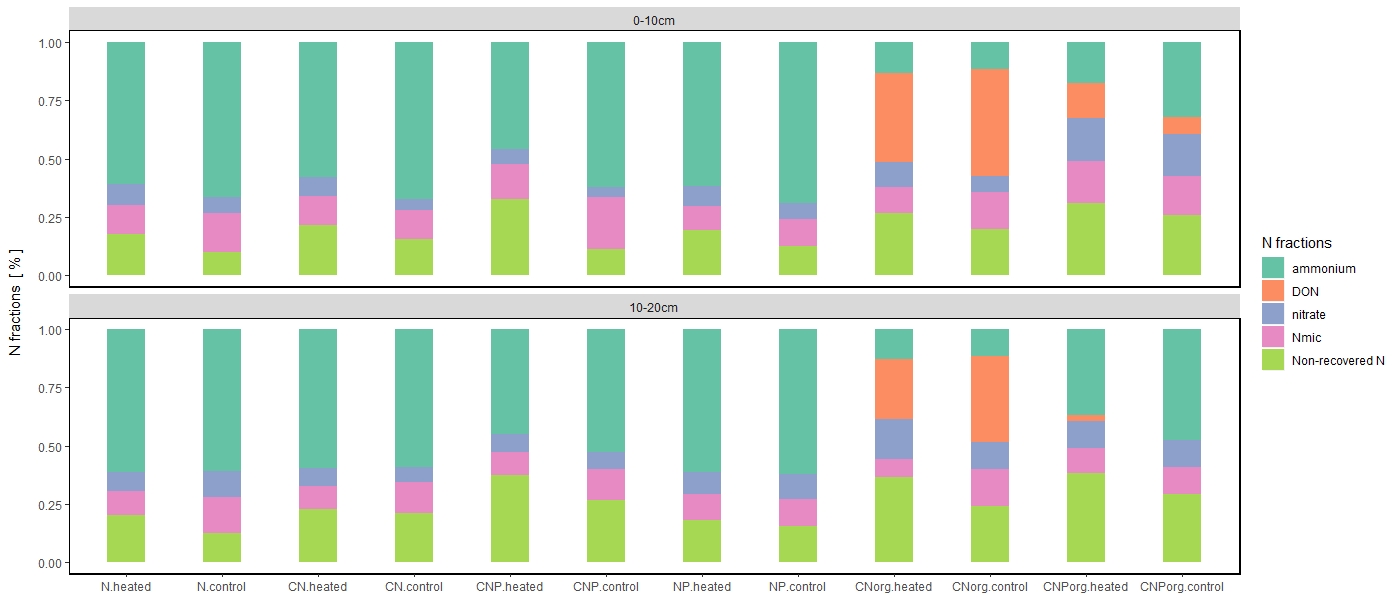


**Figure S2:** Labile soil N fractions expressed as a percentage of total labile soil N 24h after substrate addition at two mean field temperatures (control soils: 16°C; heated soils: 20°C) from two soil depths (0-10 cm; 10-20 cm).


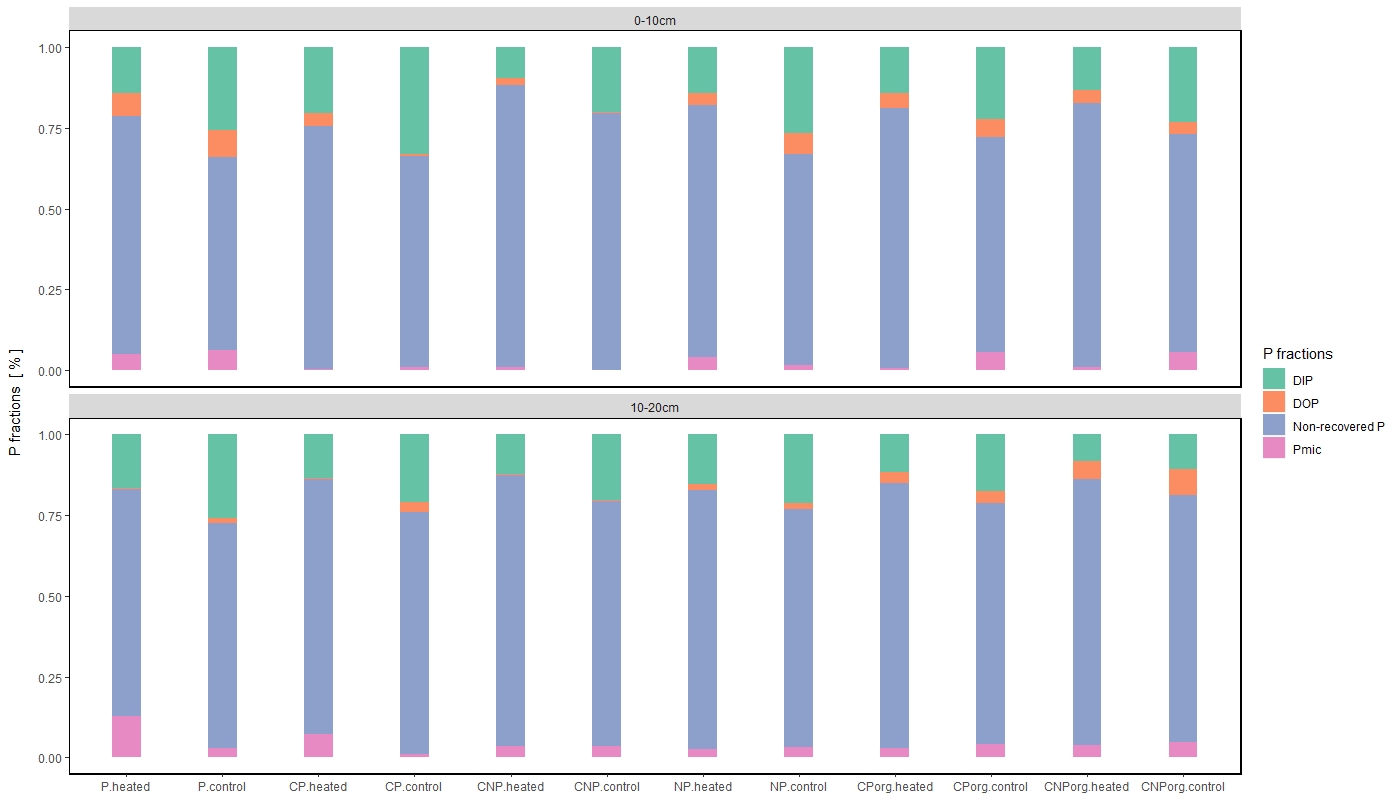


**Figure S3:** Labile soil P fractions expressed as a percentage of total labile soil P 24h after substrate addition at two mean field temperatures (control soils: 16°C; heated soils: 20°C) from two soil depths (0-10 cm; 10-20 cm).


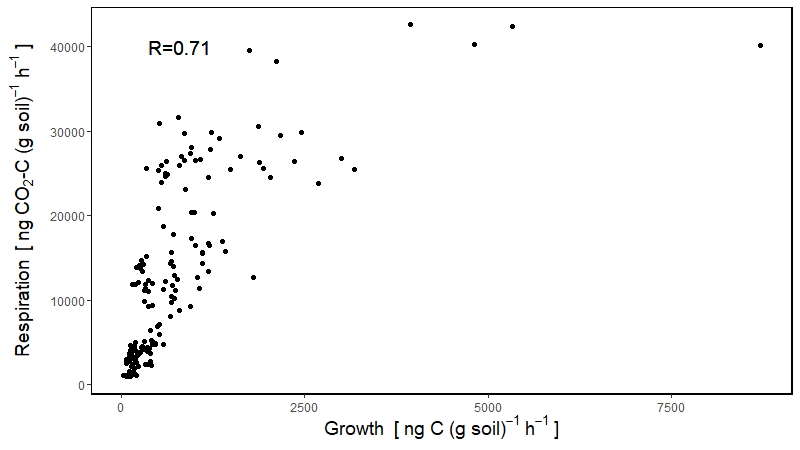


**Figure S4**: Pearson correlations between the microbial growth and the respiratory responses to substrate additions.

### **Supplementary tables**

**Table S3:** Mean ± standard deviation (n=4) of labile soil N fractions at two mean field temperatures (control soil: 16°C; heated soil: 20°C) from topsoils (0-10 cm) 24 h after nutrients were added: ammonium, nitrate, microbial biomass nitrogen (N_mic_) and dissolved organic nitrogen (DON) in µg N g^-1^ dry weight. Significant differences compared to no addition controls are marked by asterisks (data for N_mic_ were log transformed). Levels of significance were * p < 0.05, ** p < 0.01, *** p < 0.001. (-) indicates significant negative effects of adding substrates.


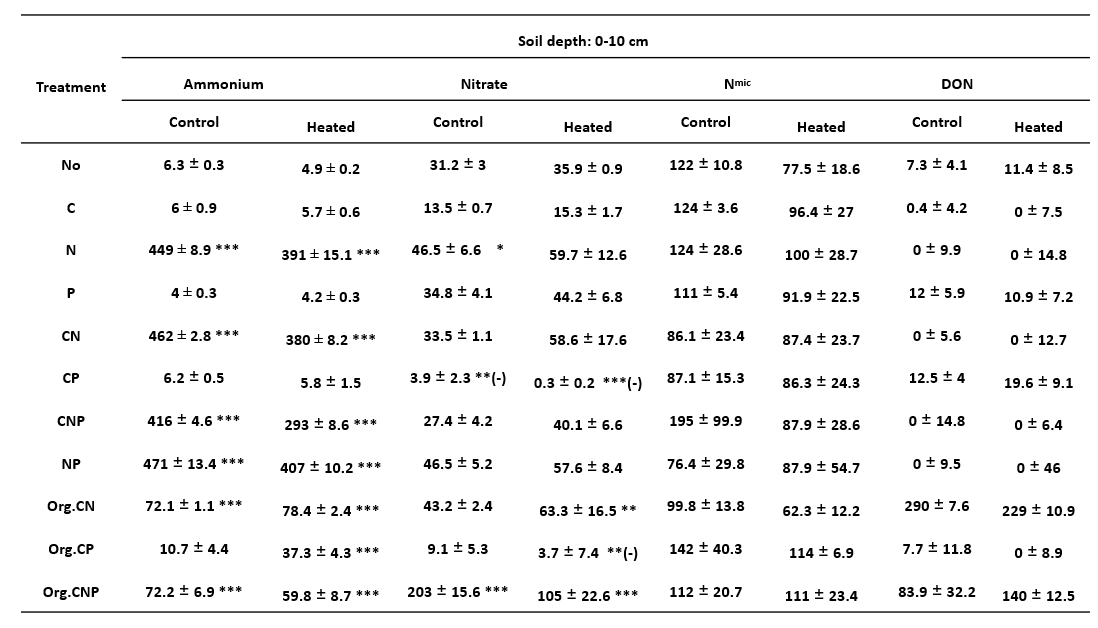


**Table S4**: Mean ± standard deviation (n=4) of labile soil N fractions at two mean field temperatures (control soil: 16°C; heated soil: 20°C) from subsoils (10-20 cm) 24 h after nutrients were added: ammonium, nitrate, microbial biomass nitrogen (N_mic_) and dissolved organic nitrogen (DON) in µg N g^-1^ dry weight. Significant differences compared to no addition controls are marked by asterisks (data for N_mic_ were log transformed). Levels of significance were * p < 0.05, ** p < 0.01, *** p < 0.001. (-) indicates significant negative effects of adding substrates.


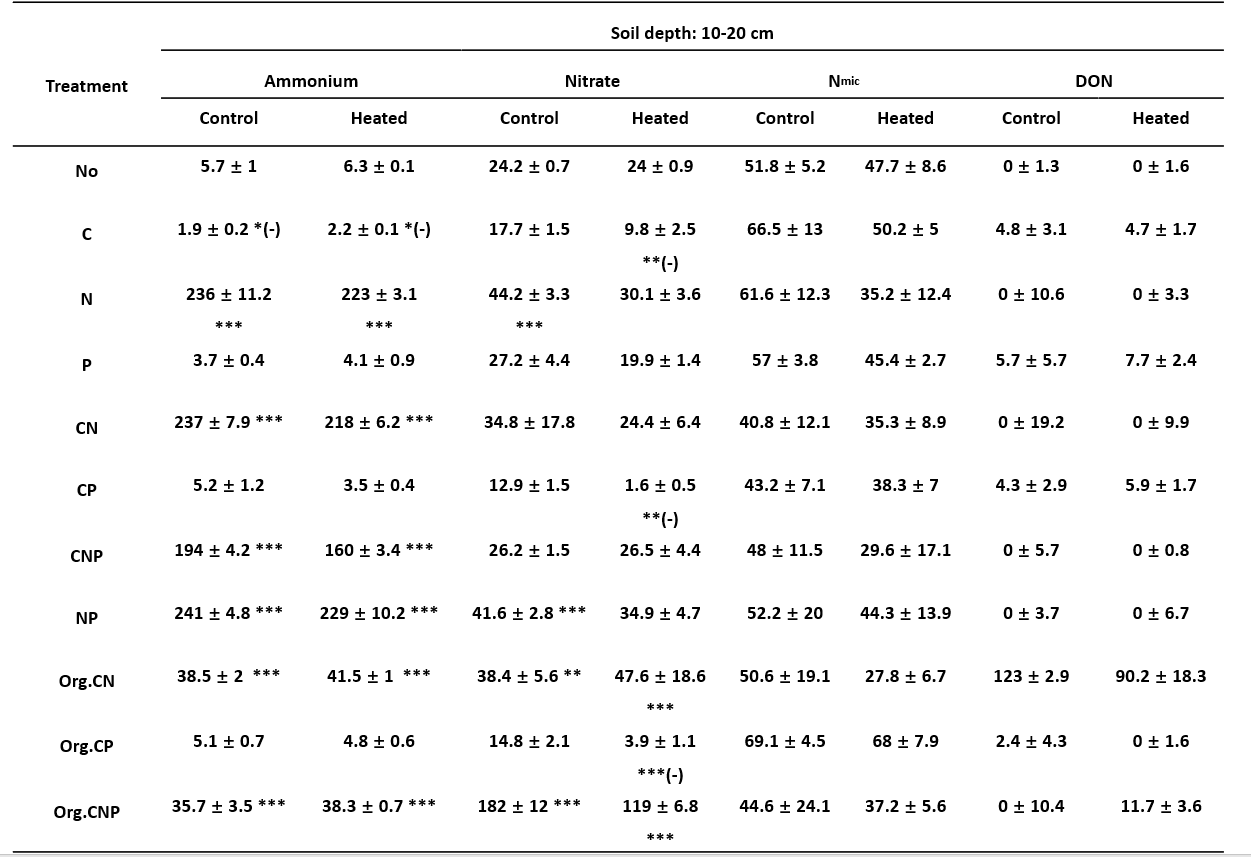


**Table S5:** Mean ± standard deviation (n=4) of labile soil P fractions at two mean field temperatures (control soil: 16°C; heated soil: 20°C) from topsoils (0-10 cm) 24 h after nutrients were added: dissolved inorganic phosphorus (DIP), organic phosphorous (DOP) and microbial biomass phosphorous (P_mic_) in µg P g^-1^ dry weight. Significant differences compared to no addition controls are marked by asterisks (data for P_mic_ and phosphate were sqrt and log transformed, respectively). Levels of significance were * p < 0.05, ** p < 0.01, *** p < 0.001.


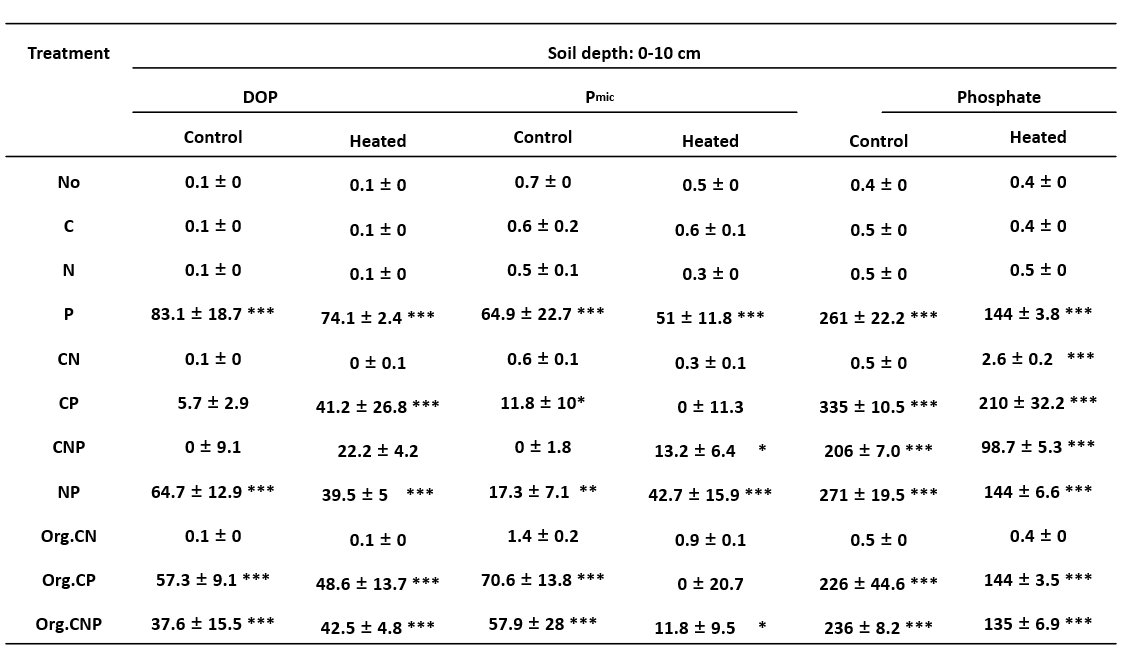


**Table S6**: Mean ± standard deviation (n=4) of labile soil P fractions at two mean field temperatures (control soil: 16°C; heated soil: 20°C) from subsoils (10-20 cm) 24 h after nutrients were added: dissolved inorganic phosphorus (DIP), organic phosphorous (DOP) and microbial biomass phosphorous (P_mic_) in µg P g^-1^ dry weight. Significant differences compared to no addition controls are marked by asterisks (data for P_mic_ and phosphate were sqrt and log transformed, respectively). Levels of significance were * p < 0.05, ** p < 0.01, *** p < 0.001.


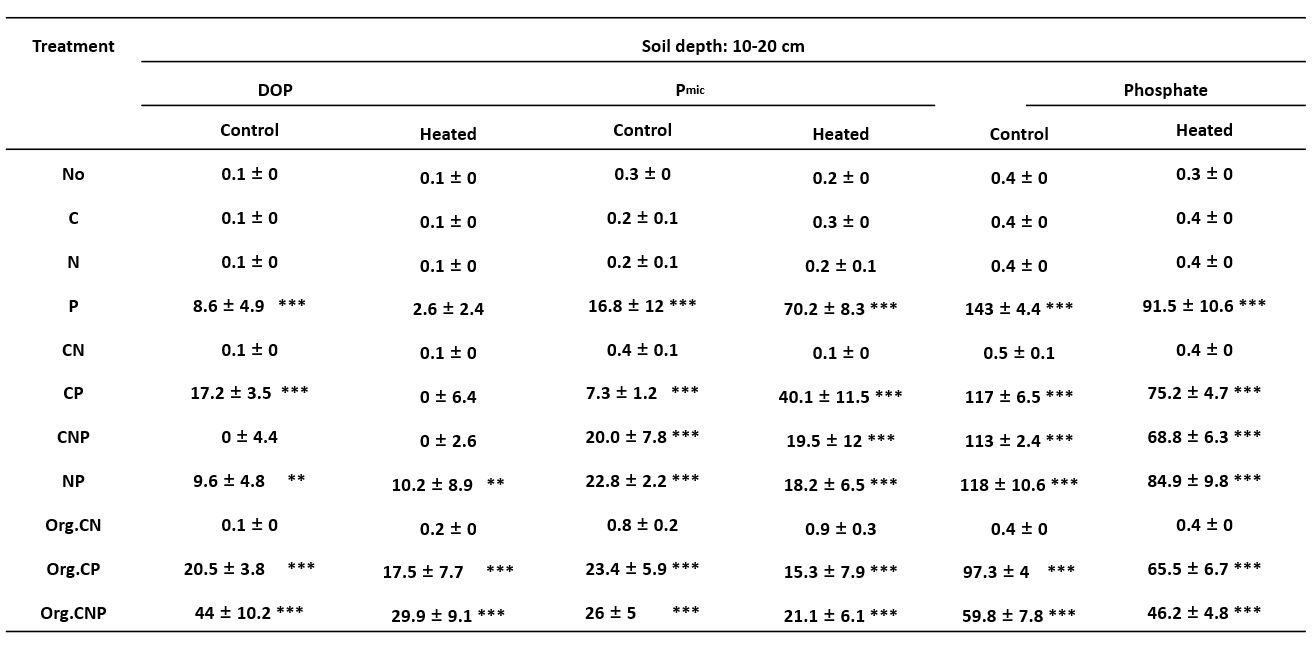


**Table S7**: Mean ± standard deviation (n=4) of labile soil C fractions at two mean field temperatures (control soil: 16°C; heated soil: 20°C) from two soil depths (0-10 cm; 10-20 cm ) 24 h after nutrients were added: microbial biomass C (C_mic_) and dissolved organic carbon (DOC) in µg C g^-1^ dry weight. Significant differences compared to no addition controls are marked by asterisks (data for C_mic_ were log transformed). Levels of significance were * p < 0.05, ** p < 0.01, *** p < 0.001.


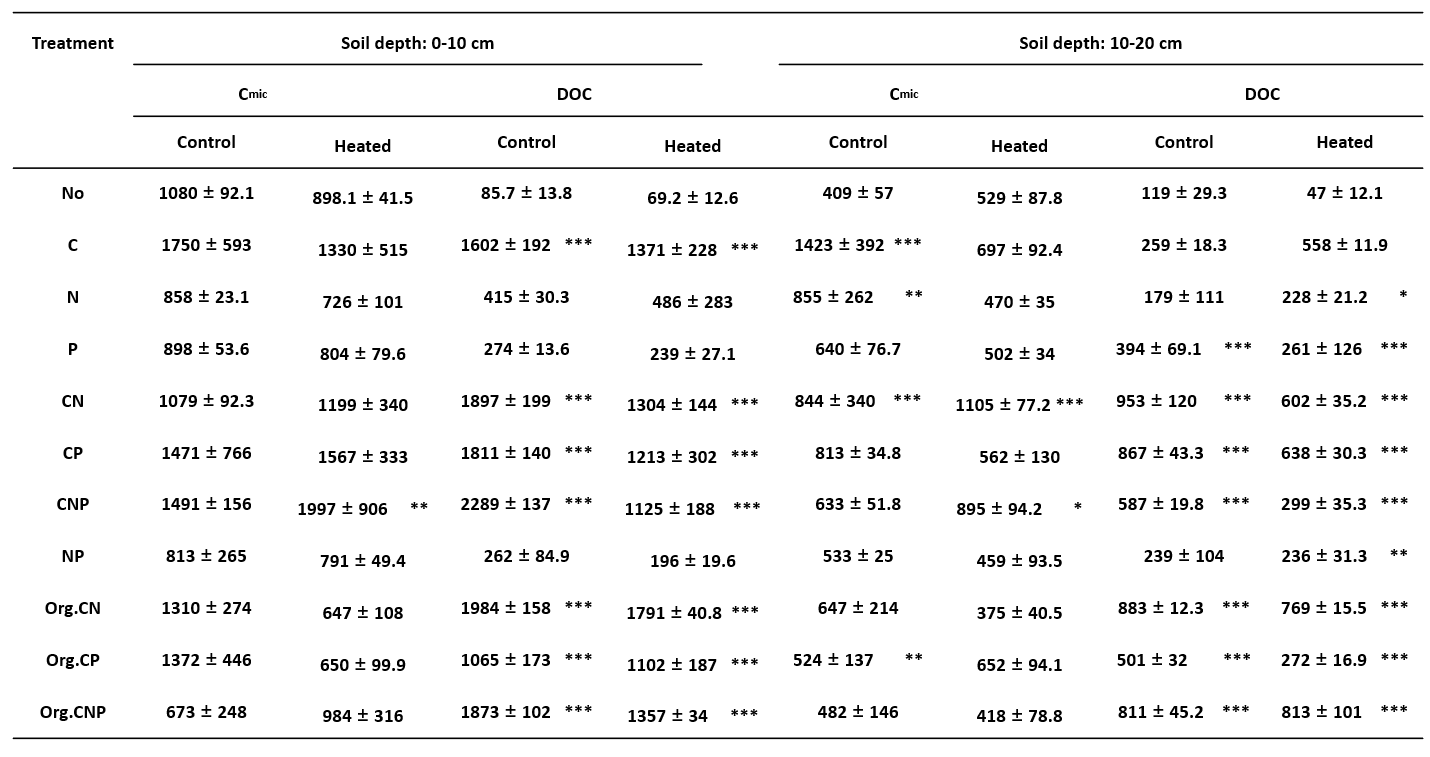


**Table S8**: Microbial biomass C in µg C g^-1^ dry weight, N in µg N g^-1^ dry weight and P in µg P g^-1^ dry weight at two mean field temperatures (control soils: 16°C, heated soils: 20°C) from topsoils (0-10 cm) 24h after substrate addition. Values are given as mean with standard deviation (n=4), and significant differences compared to no addition controls are marked by asterisks. Levels of significance were * p < 0.05, ** p < 0.01, *** p < 0.001.


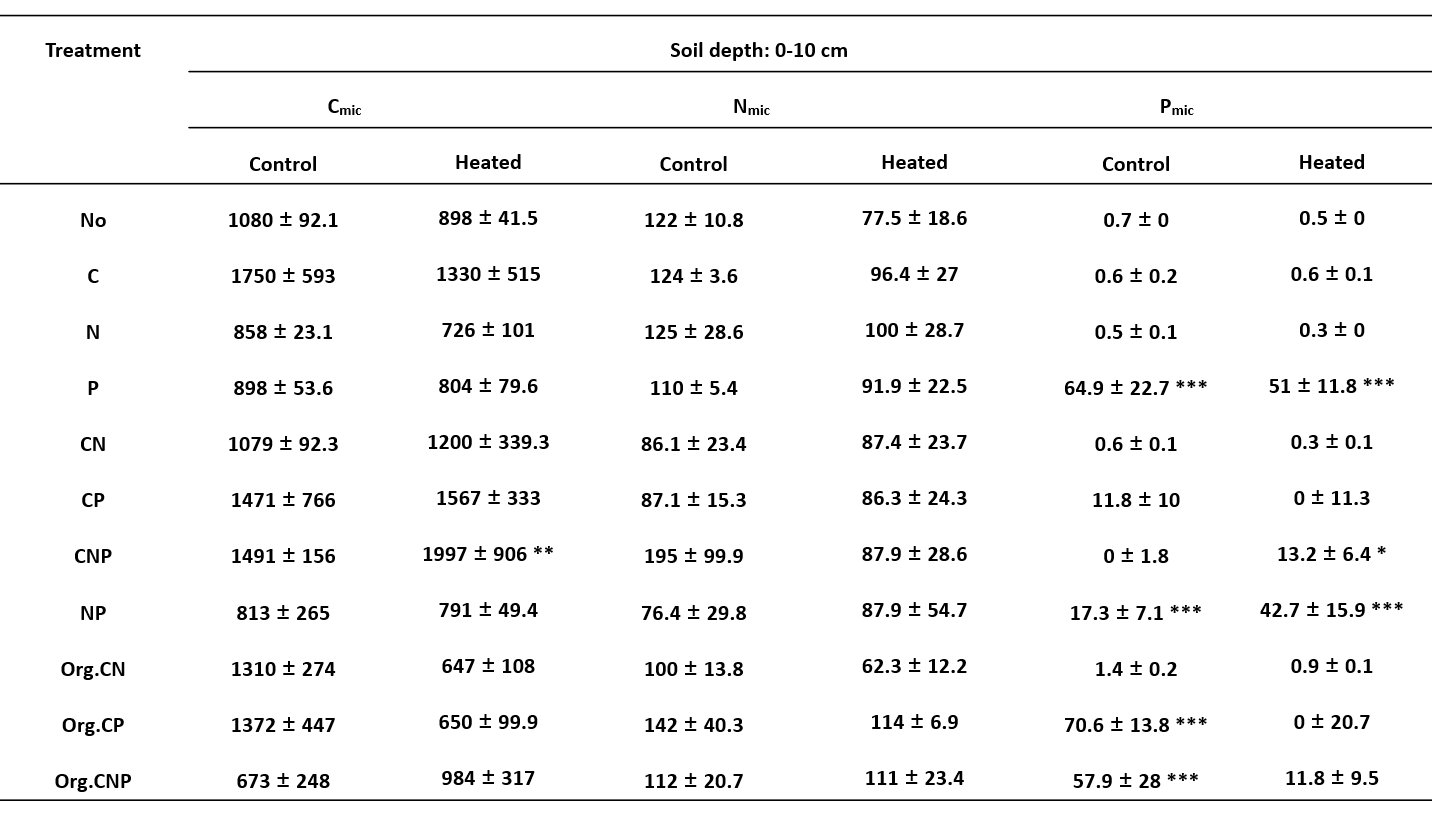


**Table S9**: Microbial biomass C in µg C g^-1^ dry weight, N in µg N g^-1^ dry weight and P in µg P g^-1^ dry weight at two mean field temperatures (control soils: 16°C, heated soils: 20°C) from subsoils (10-20 cm) 24h after substrate addition. Values are given as mean with standard deviation (n=4), and significant differences compared to no addition controls are marked by asterisks. Levels of significance were * p < 0.05, ** p < 0.01, *** p < 0.001.


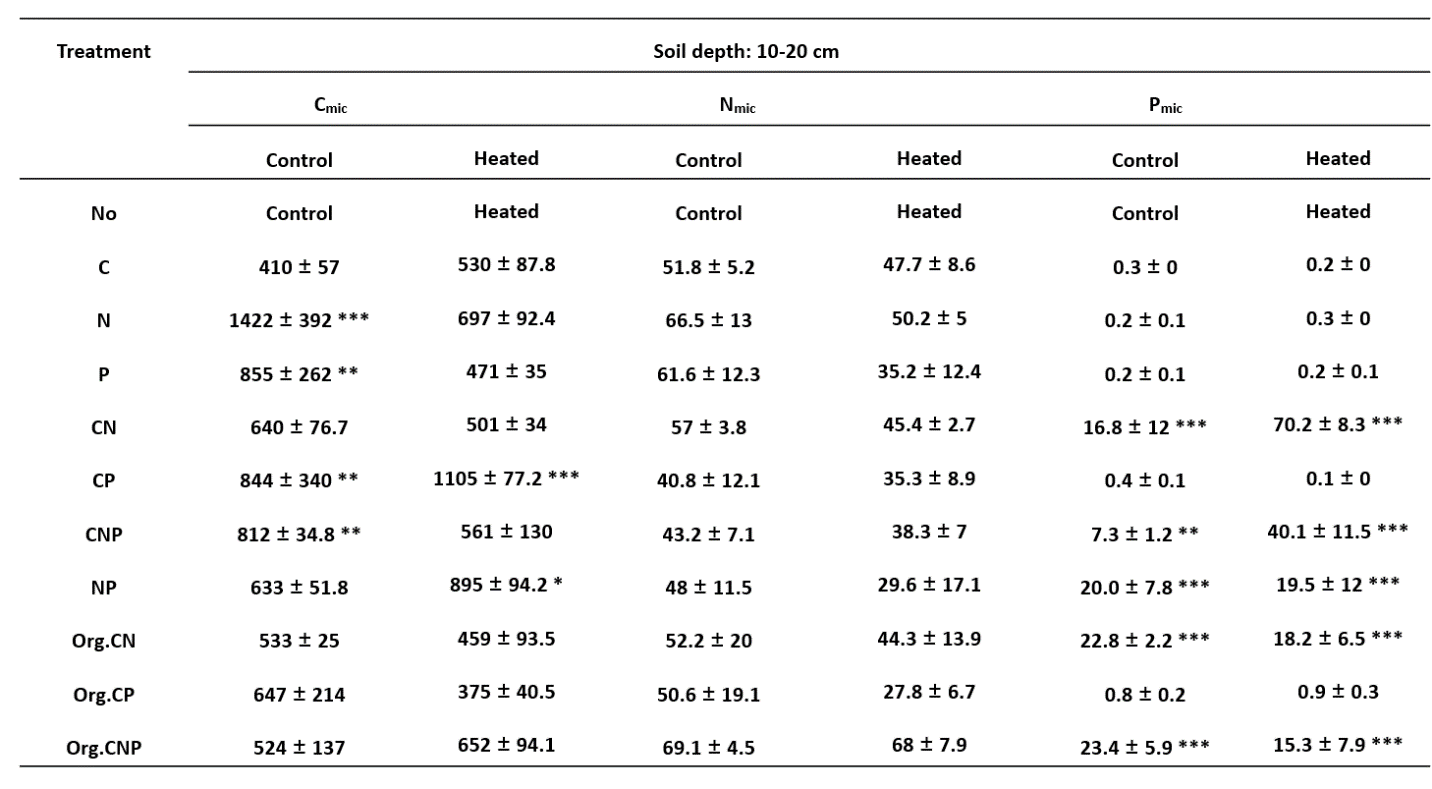


**Table S10:** Molar ratios of microbial biomass C:N, C:P and N:P at two mean field temperatures (control soils: 16°C; heated soils: 20°C) from topsoils (0-10 cm) 24 h after substrate addition. Values are given as mean with standard deviation (n=4), and significant differences compared to no addition controls are marked by asterisks. Levels of significance were * p < 0.05, ** p < 0.01, *** p < 0.001.


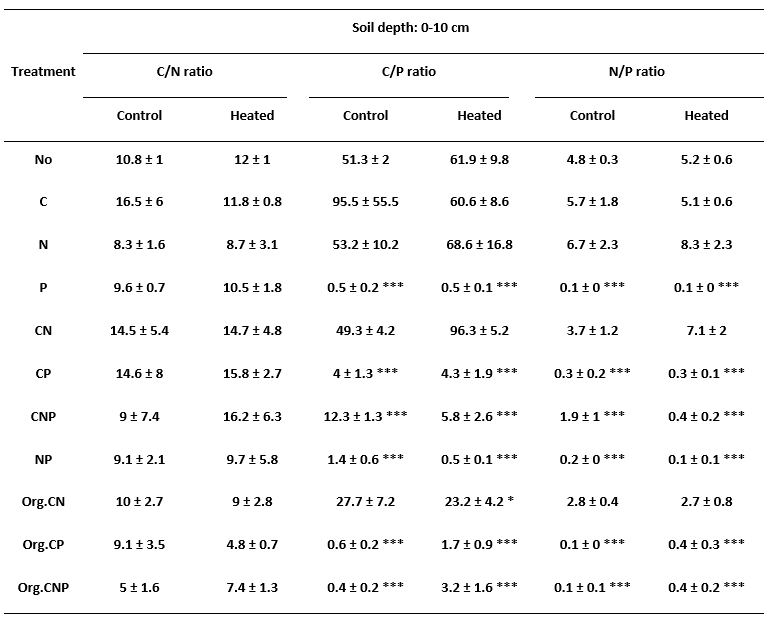


**Table S11:** Molar ratios of microbial biomass C:N, C:P and N:P at two mean field temperatures (control soils: 16°C; heated soils: 20°C) from subsoils (10-20 cm) 24 h after substrate addition. Values are given as mean with standard deviation (n=4), and significant differences compared to no addition controls are marked by asterisks. Levels of significance were * p < 0.05, ** p < 0.01, *** p < 0.001.


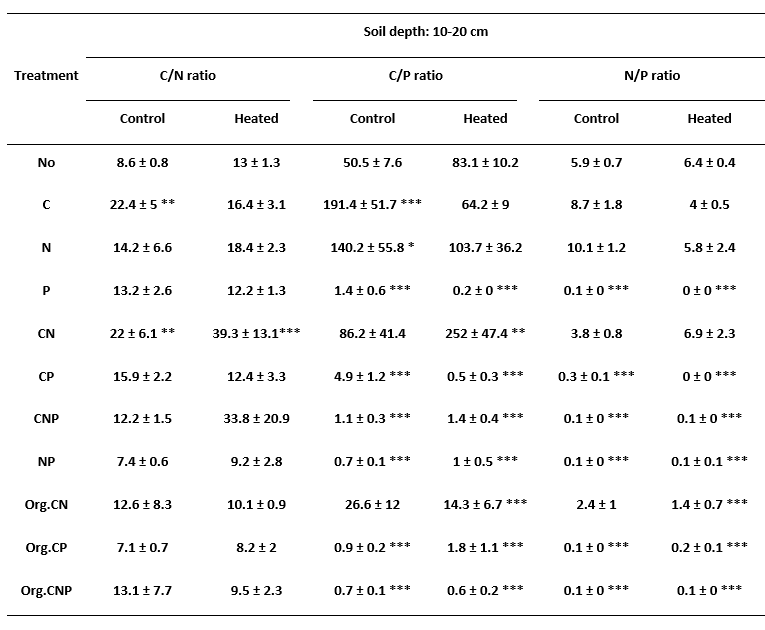


**Table S12**: P and F values of 5-way ANOVA test. Data was log transformed to fit for normal distribution of residual and homogeneity of variance. Main effects of single factors and their interactive effects are displayed for C: carbon, N: nitrogen, P: phosphorous, warming and soil depth. Significance levels: ***. P < 0.001; **. P < 0.01; *. P < 0.05; n.s. not significant.

|  |  | **Microbial growth** | **Microbial respiration** | **Microbial C uptake** |
| --- | --- | --- | --- | --- |
| **C** | **P** | ******* | ******* | ******* |
|  | **F** | **858** | **21724** | **22038** |
| **N** | **P** | ***** | ******* | ******* |
|  | **F** | **6.9** | **48.2** | **34.0** |
| **P** | **P** | ******* | ******* | ******* |
|  | **F** | **171** | **1147** | **1214** |
| **Warming** | **P** | ***** | ******* | ******* |
|  | **F** | **6.6** | **42.5** | **53.9** |
| **Depth** | **P** | ******* | ******* | ******* |
|  | **F** | **44.8** | **99.8** | **145** |
| **C x N** | **P** | ******* | **n.s** | **n.s** |
|  | **F** | **37.7** | **0.2** | **1.6** |
| **C x P** | **P** | ******* | ******* | ******* |
|  | **F** | **49.1** | **140** | **99.7** |
| **C x N x P** | **P** | ******* | ******* | ******* |
|  | **F** | **35.4** | **30.1** | **40.8** |
| **N x P** | **P** | ****** | ***** | **n.s** |
|  | **F** | **10.6** | **5.1** | **0.2** |
| **C x Warming** | **P** | ***** | ****** | ******* |
|  | **F** | **4.4** | **9.8** | **13.8** |
| **N x Warming** | **P** | ******* | **n.s** | **n.s** |
|  | **F** | **15.3** | **0.0** | **0.6** |
| **P x Warming** | **P** | ******* | ******* | ******* |
|  | **F** | **26.7** | **20.5** | **30.0** |
| **C x N x Warming** | **P** | ****** | ***** | ***** |
|  | **F** | **7.4** | **4.2** | **6.5** |
| **C x P x Warming** | **P** | ******* | ***** | ****** |
|  | **F** | **19.5** | **4.4** | **8.8** |
| **C x N x P x Warming** | **P** | **n.s** | ****** | ****** |
|  | **F** | **0.1** | **8.7** | **9.8** |
| **C x Depth** | **P** | ***** | **n.s** | **n.s** |
|  | **F** | **6.0** | **0.9** | **1.8** |
| **N x Depth** | **P** | **n.s** | **n.s** | **n.s** |
|  | **F** | **0.9** | **0.1** | **0.6** |
| **P x Depth** | **P** | **n.s** | ******* | ******* |
|  | **F** | **0.4** | **89.3** | **77.7** |
| **C x N x Depth** | **P** | **n.s** | **n.s** | **n.s** |
|  | **F** | **0.7** | **0.1** | **0.9** |
| **C x P x Depth** | **P** | ****** | ******* | ****** |
|  | **F** | **9.0** | **13.4** | **10.2** |
| **C x N x P x Depth** | **P** | ******* | **n.s** | **n.s** |
|  | **F** | **16.6** | **1.3** | **0.1** |
| **Warming x Depth** | **P** | ******* | **n.s** | ******* |
|  | **F** | **129** | **0.0** | **13.1** |

**Table S13:** P and F values of 6-way ANOVA test. Data was log transformed to fit for normal distribution of residual and homogeneity of variance. Main effects of single factors and their interactive effects are displayed for CN: substrate addition containing C and N addition, CP: substrate addition containing C and P addition, CNP: substrate addition containing C, N and P, warming, depth and substrate quality: organic/inorganic. Significance levels: ***. P < 0.001; **. P < 0.01; *. P < 0.05; n.s. not significant.

|  |  | **Microbial growth** | **Microbial respiration** | **Microbial C uptake** |
| --- | --- | --- | --- | --- |
| **CN** | **P** | ******* | ******* | ******* |
|  | **F** | **219** | **52.0** | **89.3** |
| **CP** | **P** | ******* | ******* | ******* |
|  | **F** | **39.9** | **1940** | **1817** |
| **CNP** | **P** | ******* | ******* | ******* |
|  | **F** | **268** | **4109** | **4046** |
| **Warming** | **P** | ****** | ******* | ******* |
|  | **F** | **8.4** | **42.0** | **52.4** |
| **organic/inorganic** | **P** | ******* | ******* | ******* |
|  | **F** | **193** | **5563** | **5450** |
| **Depth** | **P** | ******* | ******* | ******* |
|  | **F** | **35.2** | **33.3** | **51.9** |
| **CN x Warming** | **P** | ******* | ***** | ****** |
|  | **F** | **27.1** | **5.1** | **9.5** |
| **CP x Warming** | **P** | ****** | **n.s** | ***** |
|  | **F** | **11.2** | **2.6** | **5.2** |
| **CNP x Warming** | **P** | ***** | ****** | ******* |
|  | **F** | **6.2** | **10.7** | **14.3** |
| **CN x Depth** | **P** | **n.s** | ******* | ******* |
|  | **F** | **3.5** | **28.3** | **27.2** |
| **CP x Depth** | **P** | **n.s** | **n.s** | **n.s** |
|  | **F** | **1.1** | **2.0** | **3.3** |
| **CNP x Depth** | **P** | **n.s** | ***** | ***** |
|  | **F** | **2.9** | **4.1** | **5.9** |
| **CN x organic/inorganic** | **P** | **n.s** | ******* | ******* |
|  | **F** | **0.0** | **100.5** | **94.5** |
| **CP x organic/inorganic** | **P** | ******* | ******* | ******* |
|  | **F** | **70.6** | **492** | **522** |
| **CNP x organic/inorganic** | **P** | **-** | **-** | **-** |
|  | **F** | **-** | **-** | **-** |
| **Warming x Depth** | **P** | ******* | **n.s** | ******* |
|  | **F** | **75.5** | **3.0** | **19.0** |
| **Warming x organic/inorganic** | **P** | ******* | ****** | ******* |
|  | **F** | **27.7** | **10.1** | **16.9** |
| **Depth x organic/inorganic** | **P** | ***** | ******* | ******* |
|  | **F** | **4.5** | **17.4** | **19.0** |

**Table S14**: Measurements of microbial growth rate (ng C (g soil)^-1^ h^-1^), microbial C respiration (ng C (g soil)^-1^ h^-1^) and microbial C uptake (ng C (g soil)^-1^ h^-1^) at two mean field temperatures (control soils: 16°C; heated soils: 20°C) from two soil depths (0-10cm; 10-20 cm) 24 h after labile C, N and P addition.
